# Pressure Dependence of Rate Coefficients of Unimolecular and Chemical Activation Reactions Connected to the Potential Energy Wells of Si_2_H_2_Cl_4_, Si_2_Cl_6_, and Si_2_Cl_4_ via Rice–Ramsperger–Kassel–Marcus Calculations

**DOI:** 10.1021/acs.jpca.2c06195

**Published:** 2022-11-13

**Authors:** Kaito Noda, Yoshihiro Jagawa, Akio Fuwa, Nílson Kunioshi

**Affiliations:** † Waseda University, 3-4-1 Ohkubo, Shinjuku, Tokyo169-8555, Japan; ‡ SUMCO Corporation, 1-52 Kubara, Yamashiro-cho, Imari-shi, Saga849-4256, Japan

## Abstract

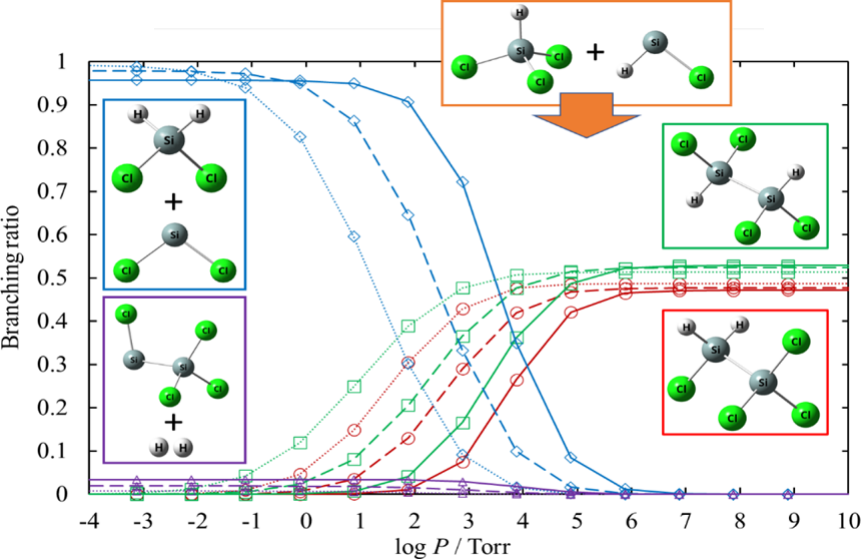

Rate coefficients for elementary reactions connected to the potential energy wells of Si_2_H_2_Cl_4_, Si_2_Cl_6_, and Si_2_Cl_4_, which are important Si_2_ species in chemical vapor deposition (CVD) processes that use chlorosilanes as silicon source gases, were determined through the Rice–Ramsperger–Kassel–Marcus theory under various conditions of temperature and pressure. The optimized structures and vibrational frequencies of the reactants, products, and transition state were obtained using (U)B3LYP/6-31+G(d,p), and the single-point energies of the optimized structures were recalculated using the coupled cluster method with single and double excitations plus triple perturbation (U)CCSD(T) with complete basis set extrapolation. Many of the unimolecular decomposition channels and chemical activation reactions investigated in this work were found to be in the fall-off regime under subatmospheric to moderately high-pressure conditions so that it is expected that accurate modeling of the gas phase in the chlorosilane CVD reactor requires careful determination of the rate coefficients as functions of temperature and pressure for the conditions of interest, instead of using high-pressure limit rate coefficients. The rate coefficients determined here were expressed through Chebyshev coefficients and also modified Arrhenius parameters to be used in simulations of systems under a wide range of temperature and pressure conditions.

## 1 Introduction

When silicon surfaces of extreme purity and perfect flatness are required, epitaxial growth through chemical vapor deposition (CVD) processes is often performed. This process is industrially important, and continuous improvement of its quality is a requirement for silicon wafers produced to meet the growing needs of the semiconductor technology.

Numerous chemical (gas-phase and surface reactions) and physical (gas convection and diffusion and thermal conduction) phenomena occur simultaneously and interact in complex ways in CVD reactors, making the elucidation and eventually optimization of such a process quite difficult.^1^ Simulation of the CVD reactor is a very useful approach for elucidating the growth process and seeking better operation conditions.

As for the elucidation of chemical reaction mechanisms, gas-phase and surface chemistry has to be analyzed in detail. Based on an extensive literature survey, our group has already built a kinetic model that was able to reproduce experimental results related to the so-called “clean conversion” process or the reaction of SiCl_4_ with H_2_ to produce SiHCl_3_ (trichlorosilane or TCS) and HCl at atmospheric pressure.^2^ Most of the elementary reactions in the model were obtained from models of gas-phase decomposition of TCS in the CVD reactor available in the literature.^3,4^

Many of the elementary reactions in this model are unimolecular decomposition or recombination reactions whose rate coefficients may depend on pressure. However, most of the rate coefficients that can be found in the literature are limited to the high pressure limit. The clean conversion process was modeled successfully probably because relatively few reactions were important, but in the CVD reactor, many more elementary reactions play important roles.

Swihart and Carr^4^ proposed that the composition among the various SiH_*x*_Cl_4–*x*_ (*x* = 1, 2, 3, and 4) species change through recombination reactions that produce disilanes (the disilane mechanism). They calculated the rate coefficients of the decomposition reactions of several chlorinated disilanes in their reaction mechanism through an ab initio study at the G2//MP2/6-31G(d,p) level of theory and conventional transition state theory (TST).^5^

Ravasio et al.^3^ performed simulations of a perfectly stirred reactor (PSR) in which TCS is used as the source gas. They revealed that the following set of reactions occur.
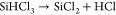
r1
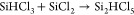
r2
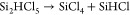
r3
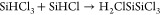
r4
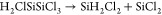
r5

They showed that the following two reactions also become more important as the concentration of HCl increases in the reactor.
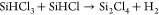
r6
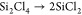
r7

Ravasio et al. also revealed that with the increase of the residence time, the radical pathway, which consists of a set of homolytic decomposition reactions and several propagation reactions (the radical mechanism), becomes dominant. The most abundant radical was SiCl_3_, and the fastest termination step of the radical chain was the recombination of two SiCl_3_ radicals.
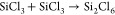
r8

These reactions, except reaction r6, are unimolecular decomposition or recombination reactions and their rate coefficients are expected to have pressure dependence. The rate coefficients of gas-phase reactions in their kinetic mechanism were calculated at the CCSD(T)/CBS//B3LYP/6-31+G(d,p) level of theory and TST.

We have already analyzed the pressure dependence of reactions of formation and dissociation of SiHCl_3_, SiH_2_Cl_2_, SiH_3_Cl, SiHCl_2_, and Si_2_HCl_5_,^6,7^ including reactions r1, r2, and r3, and confirmed that the rate coefficients of many channels (dissociation channels and chemical activation reactions) are in the fall-off region under low to moderate pressure, indicating that the adoption of high-pressure-limit rate coefficients in simulation of reactions at atmospheric pressure may lead to inaccurate results. In addition, it was shown that the pressure dependence of many other reactions should be verified in order to build a kinetic model that can be used in a wide range of pressure and temperature conditions. In this work, we show the temperature and pressure dependence of rate coefficients for the unimolecular dissociations of Si_2_H_2_Cl_4_, Si_2_Cl_6_, and Si_2_Cl_4_ and the related recombination and chemical activated reactions SiHCl_3_ + SiHCl (r4), SiH_2_Cl_2_ + SiCl_2_ (−r5), SiCl_3_ + SiCl_3_ (r8), and SiCl_3_ + SiCl. Reaction r6 was calculated as a bimolecular reaction having a rate coefficient independent of pressure in Ravasio et al.’s work, but in the present work, it is treated as a chemical activation reaction in the Si_2_H_2_Cl_4_ potential energy well.

## 2 Computational Details

The elementary reactions analyzed in this work are those related to the potential energy wells of Si_2_H_2_Cl_4_, Si_2_Cl_6_, and Si_2_Cl_4_. Each channel links the bottom of a well (single molecule) to a pair of decomposed molecules or to an isomer. Gaussian09^8^ was used in the quantum chemical calculations. The structure optimizations of all stationary points and their vibrational frequencies were obtained at the (U)B3LYP/6-31G+(d,p) level. The potential energies were determined at the (U)CCSD(T) level of theory and extrapolated to the infinite basis set using aug-cc-pVDZ and aug-cc-pVTZ basis sets, as proposed by Martin.^9^ This is the same approach as that used by Ravasio et al.^3^ in their analysis of the gas-phase reactions of chlorosilanes. The potential energy surfaces of reactions not having a saddle point were scanned through constrained energy minimizations along the length of the breaking bond at the same level of theory. This scanning may converge on inappropriate potential energy surfaces such as excited states. Therefore, we confirmed that the values of the potential energy or vibrational frequencies vary smoothly from the values at the reactants to the asymptotic value at the products as the breaking bond length increases. These values were obtained at the UCCSD(T)/CBS//UB3LYP/6-31+G(d,p) level. Although CCSD(T) is a single-reference based method, Walch and Dateo showed that accurate energetics of the reactions of SiCl_2_ and SiHCl with H and Cl atoms can be obtained by using CCSD(T).^10^ Zhang et al.^11^ also adapted the single-reference calculation method CCSD(T)/jul-CBS//B2PLYP/may-cc-pVTZ and found it to be an appropriate level for the unimolecular decomposition of SiHCl_*x*_ (*x* = 1, 2, and 3) including Si–H or Si–Cl bond breaking reactions. In our previous work,^6^ we also studied the breaking bond reactions using GAMESS at the multi-reference CR-CC (2,3)/CBS//UB3LYP/6-31+G(d,p) level of theory^12^ but found discontinuity in energy as the bond length increased for some of the reactions and virtually no difference from the results obtained at the UCCSD(T)/CBS//UB3LYP/6-31+G(d,p) for other reactions. In the CCSD(T) calculations, we performed the T1 diagnostic to the relevant reactions for verifying the quality of single-reference correlation.^13^ The T1 values for the CCSD(T) results exceeded 0.04 in a few cases. The T1 value also changed smoothly as the breaking bond length increased in all bond breaking reactions. This value is greater than the suggested 0.02 threshold,^13^ but from general considerations including continuity in energy variation with the bond length increase, we concluded that the CCSD(T) results are more reliable than those obtained through CR-CC.

The rate coefficients in the high pressure limit (HPL) of the channels that have tight transition states (TSs) were calculated through the conventional TST and those of the channels not having a saddle point were calculated through the variational TST (VTST)^14^ to compare with the high-pressure-limit rate coefficients of the previous studies.^3,4,15−17^ One-dimensional asymmetric Eckart tunneling corrections are taken into account in the conventional TST rate coefficient calculations using the GPOP program.^18^

In this paper, the torsional motions at the Si–Si bond of most of the Si_2_ species and some TSs are treated as a sinusoidally hindered internal rotor with a potential energy curve
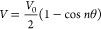
1where *V*_0_ is the potential barrier height, *n* is the symmetry number of the internal rotation, and θ is the angle of the rotation. Its partition function and thermodynamic function are calculated by using the Pitzer–Gwinn approximation.^19^ The value of the potential barrier height *V*_0_ is determined so that the partition function closely agrees with the exact partition function. To calculate the exact partition function, the potential energy surfaces are examined for the internal rotations in the reactants and TSs that have a torsional motion by scanning a dihedral angle from 0 to 360° with a 10° increment at the B3LYP/6-31+G (d, p) level. The eigenstate energies and the exact partition function of the hindered rotation are obtained by solving the one-dimensional time-independent Schrödinger equation using the BEx1D program.^20^

For most of the simple bond fission reactions, which do not have a saddle point, the value of *V*_0_ is determined so that the partition function closely agrees with the partition function interpolated using the following formula,^21^ instead of the exact partition function
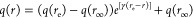
2where *r*_e_ is the Si–Si bond length of the reactant and the parameter γ is 0.75 Å. Equation 2 could not be applied in calculating the hindered rotor partition function of the simple bond fission reaction of Cl_2_SiSiCl_2_, so the exact partition functions for each reaction coordinate are calculated. The hindered rotor analysis is discussed in detail in Section 3.7.

The rate coefficients were estimated in the range of temperature from 873 to 1373 K at intervals of 50 K, at the wide range of pressure from 1 × 10^–7^ to 1 × 10^12^ atm (7.6 × 10^–5^ to 7.6 × 10^14^ Torr) and the high pressure limit, through Rice–Ramsperger–Kassel–Marcus (RRKM) theory using GPOP^18^ and SSUMES^22^ program suites, which include implementations to the UNIMOL program suite.^23^ The SSUMES program suite was used also to obtain the steady-state solution of the multichannel master equation. H_2_ was adopted as the buffer gas because we are interested in conditions related to CVD processes, where H_2_ is usually present in large excess. The required Lennard-Jones collision diameter σ and potential depth ε/*k*_B_ for each species were obtained from data distributed with CHEMKIN.^24^

As in our previous work,^6,7^ for the solution of the master equation, the average downward energy transfer per collision, ⟨Δ*E*_down_⟩, was estimated from the two parameters model, eq 3.^25^ Also, we adopted 1000 K as the reference temperature, as proposed by Miyoshi.^26^
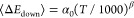
3

In eq 3, α_0_ is the value of ⟨Δ*E*_down_⟩ at 1000 K. This value was determined through the biased random walk model of Lim and Gilbert^27^ implemented in the BRW application of the UNIMOL program suite.^22^ In the biased random walk model, the Lennard-Jones parameters of the reactants of unimolecular decomposition reactions, as well as those of the elements they contain, and of the bath gas are necessary. The value of β was determined by fitting eq 3 to 12 values of ⟨Δ*E*_down_⟩ (11 calculated in the range *T* = 873 K to *T* = 1373 K with 50 K intervals, and the value at 1000 K). ⟨Δ*E*_down_⟩ was then used in SSUMES to calculate the energy transfer probability, through the exponential-down model.^14^ The estimated parameters α_0_ and β are shown in Table 1. There are no experimental data of the mean downward energy transfer per collision of chlorinated silanes, but the values in Table 1 are reasonable when compared to data related to similar systems.^28,29^

**Table 1:** Parameters α_0_ and β in eq 3 for the Energy Transfer Probability in Each Potential Well

potential well	α_0_, cm^–1^	β
Cl_3_SiSiH_2_Cl	283.5	0.98
HCl_2_SiSiHCl_2_	283.5	0.98
Si_2_Cl_6_	250.4	0.96
Cl_3_SiSiCl	238.8	0.92
Cl_2_SiSiCl_2_	238.8	0.92

**Figure 1:**
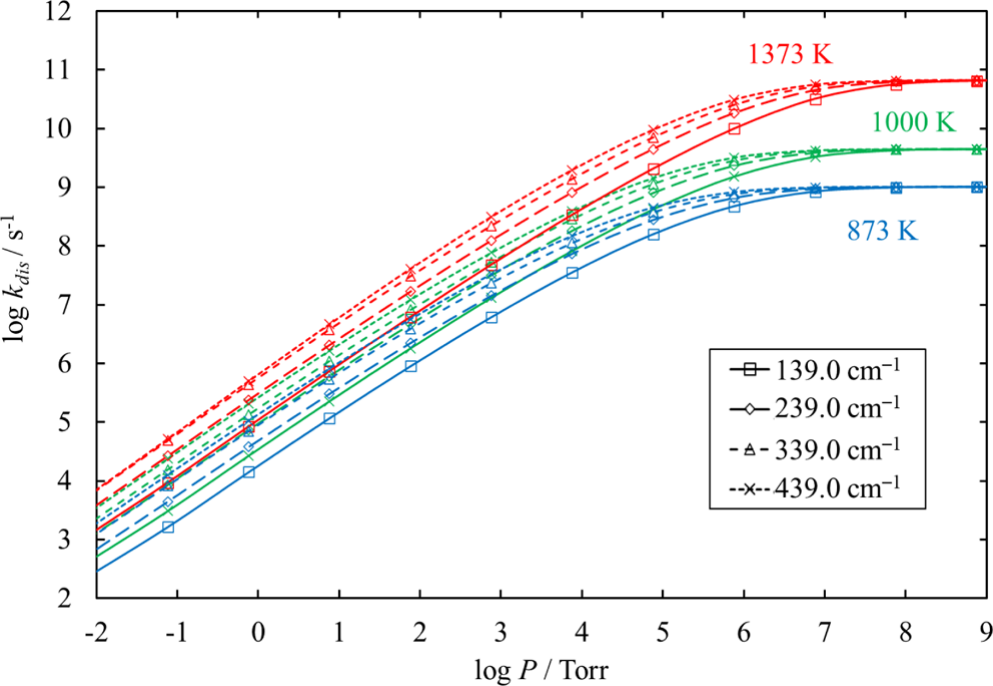
Sensitivity of the total rate coefficient for the dissociation of Si_2_Cl_4_ to α_0_ (in cm^–1^) at 873, 1000, and 1373 K.

The sensitivity of *k*_dis_, the total rate coefficients of unimolecular dissociation of Si_2_Cl_4_ estimated at 873, 1000, and 1373 K to α_0_, is shown in Figure 1. The higher the temperature and the lower the pressure, the greater the sensitivity of *k*_dis_ to α_0_. At 1373 K and 7.6 Torr (0.01 atm), the increase or decrease in α_0_ by about 40% leads to an increase or decrease in the dissociation rate coefficient by about 64 and 84%, respectively. In the case of the other chlorinated silanes, which is shown in the Supporting Information (S1), the increase or decrease in *k*_dis_ was about 40% when α_0_ increases or decreases by about 30%. Such sensitivities of *k*_dis_ to variations in α_0_ are sufficiently low.^30^ In addition, the truncation level used to cut the decomposition of the activated intermediates in the chemical activation channels was 20% of the activation energy, as proposed by Matsugi et al.^31^

## 3 Results and Discussion

### 3.1 Reaction Mechanisms

**Figure 2:**
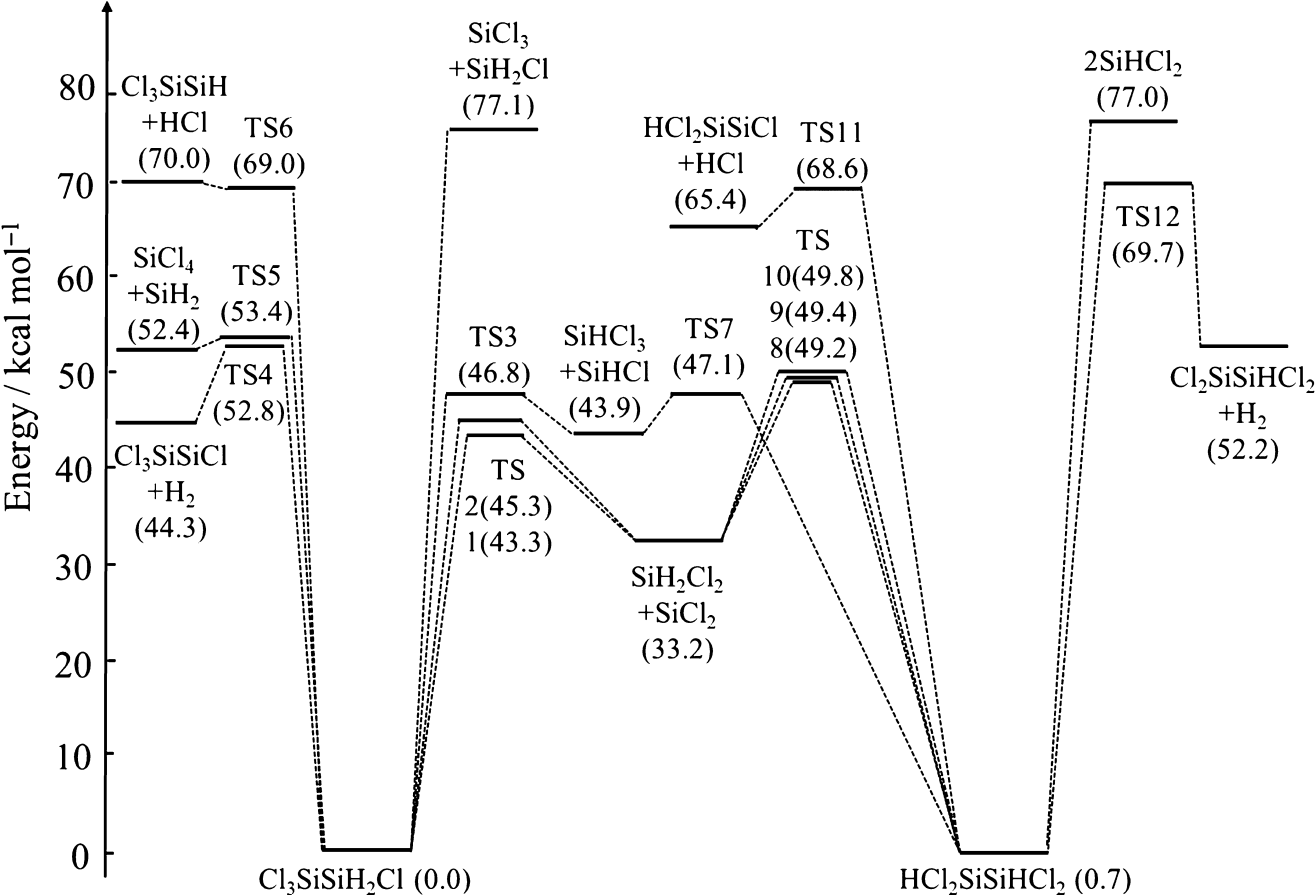
Schematic representation of the potential well of Cl_3_SiSiH_2_Cl and HCl_2_SiSiHCl_2_.

**Figure 3:**
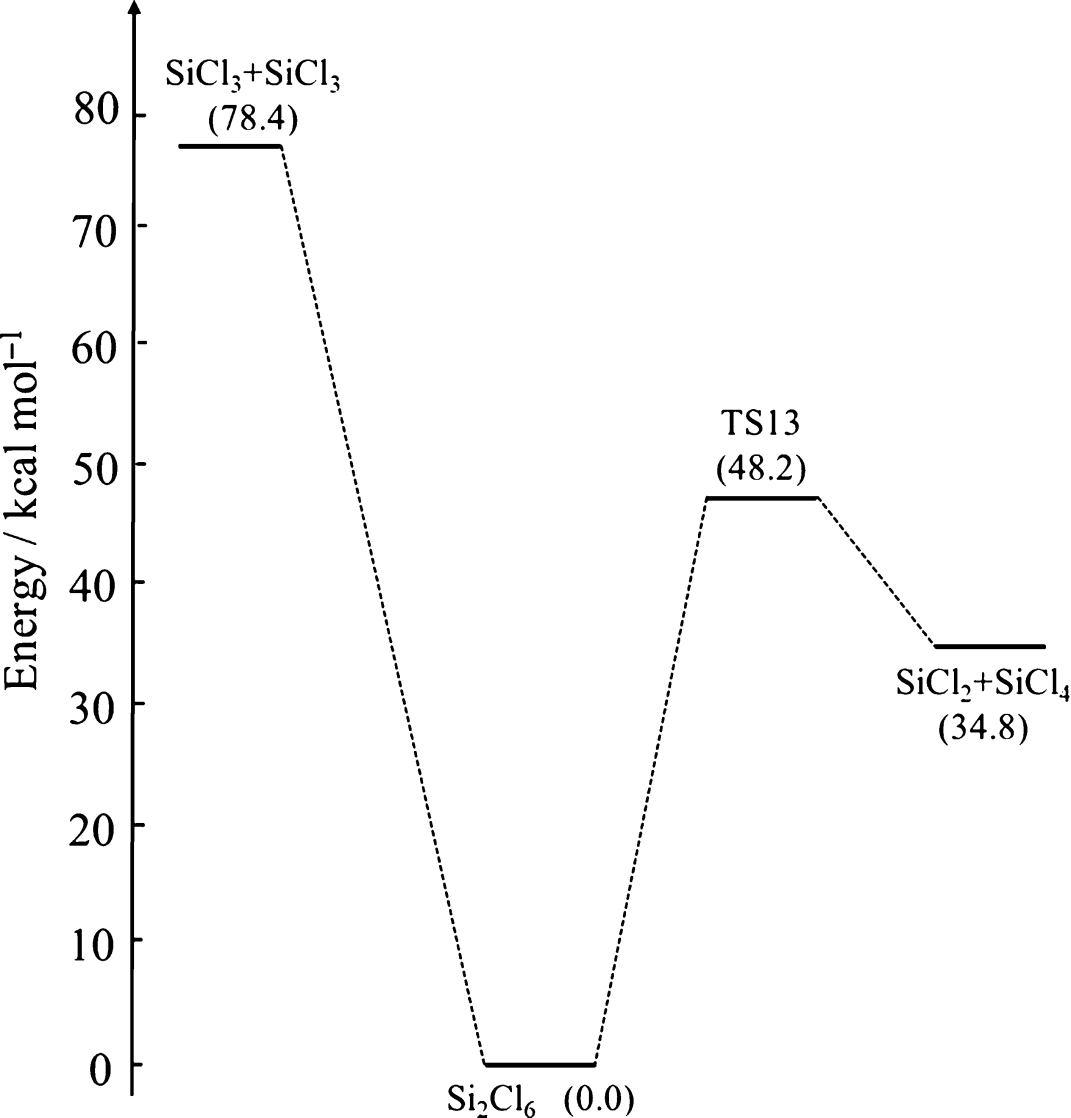
Schematic representation of the potential well of Si_2_Cl_6_.

**Figure 4:**
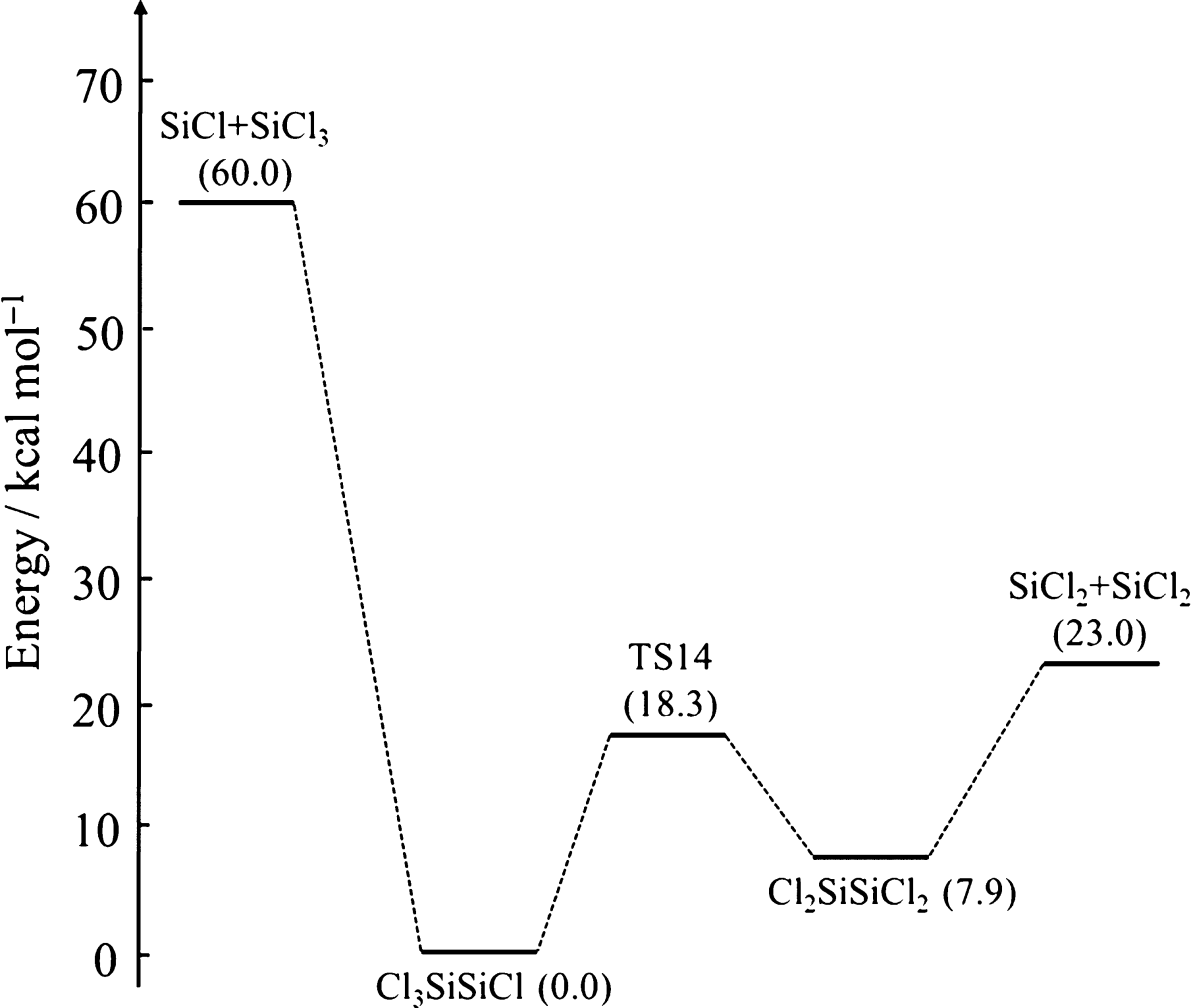
Schematic representation of the potential well of Si_2_Cl_4_.

The potential energy wells of Si_2_H_2_Cl_4_, Si_2_Cl_6_, and Si_2_Cl_4_ are shown schematically in Figures 2–4, respectively. As described in the previous section, the structures of the stationary points were optimized at the (U)B3LYP/6-31G+(d,p) level, and the potential energies were calculated at the (U)CCSD(T)/CBS level of theory. All energies in the figures include zero-point corrections and are relative to the species at the bottom of each potential energy well. The optimized structures for the stable species participating in the reactions and those for the TSs are available in the Supporting Information (S2). Their vibrational frequencies, rotational constants, symmetry numbers, and point groups are also listed in S2. In all these potential wells, channels leading to Cl_2_ formation were not found in the present work and are not reported in the literature either.

### 3.2 Si_2_H_2_Cl_4_ Potential Energy Well

Si_2_H_2_Cl_4_ has two structural isomers, Cl_3_SiSiH_2_Cl and HCl_2_SiSiHCl_2_. Cl_3_SiSiH_2_Cl can decompose as follows.
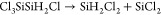
R1-1
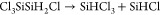
R1-2
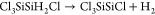
R1-3
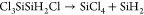
R1-4
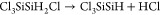
R1-5
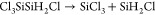
R1-6

H elimination and Cl elimination can also happen. However, they are neglected here because the reaction enthalpies are considerably high (87.9 and 106.3 kcal/mol, respectively) and the products, Cl_3_SiSiHCl, Cl_2_SiSiH_2_Cl and Cl_3_SiSiH_2_, are hardly produced in Si–H–Cl CVD. R1-1 has two pathways differentiated by the relative position of the Cl atom in the SiH_2_Cl moiety (TS1 and TS2), as already pointed out by Ravasio et al.^3^ Regarding R1-5, although the potential energy of the TS was higher than that of the products at the B3LYP level, the energy of the products was slightly higher at the CCSD(T) level. An alternative TS may exist between the TS and the products in a way similar to the results of Matsumoto et al. for the decomposition of disilane.^29^ We did not search for the alternative TS because the energy difference between the TS and the products was just 1.0 kcal/mol.

The decomposition reactions of HCl_2_SiSiHCl_2_ are
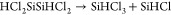
R1-7
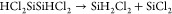
R1-8
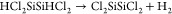
R1-9
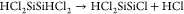
R1-10
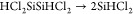
R1-11

H elimination and Cl elimination can also happen, but again, they are neglected here because the reaction enthalpies are high (87.4 and 106.2 kcal/mol, respectively) and the products, HCl_2_SiSiCl_2_, HCl_2_SiSiHCl, are hardly produced in Si–H–Cl CVD. R1-8 has three pathways differentiated by the position of the H atom in the SiHCl_2_ moiety relative to the transposing H atom (TS8, TS9, and TS10). Ravasio et al.^3^ also reported the optical isomers TS8 and TS10 but did not report on TS9. The reaction rate coefficients of the reactions that have multiple TSs are calculated as the sum of all channels.

From steric considerations, the formation of isomers such as Cl_2_SiSiHCl or Cl_3_SiSiH from either Cl_3_SiSiH_2_Cl or HCl_2_SiSiHCl_2_ were neglected: such reactions would require that one H atom and one Cl atom, each linked to different Si atoms, come together and form HCl. This is unlikely to happen because of the large distance separating H and Cl linked to different Si atoms.

### 3.3 Si_2_Cl_6_ Potential Energy Well

According to Figure 3, the dissociation channels of Si_2_Cl_6_ are:
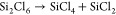
R2-1
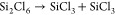
R2-2

The Cl elimination reaction can also happen but is again neglected here because its enthalpy change is too high (106.9 kcal/mol) to compete with R2-1 and R2-2.

### 3.4 Si_2_Cl_4_ Potential Energy Well

As shown in Figure 4, the isomerization and dissociation channels of Si_2_Cl_4_ are
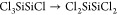
R3-1
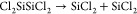
R3-2
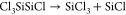
R3-3

Swihart and Carr^4^ and Ravasio et al.^3^ calculated R3-2′ (below) instead of R3-2 as the reaction in which Si_2_Cl_4_ decomposes to two SiCl_2_ because the isomerization reaction R3-1 has low activation energy and can be considered to be always in equilibrium.
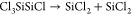
R3-2′

In this work, R3-1 and R3-2 were distinguished, and their rate coefficients were calculated because the activation energy of R3-2 was not much higher than that of −R3-1, the reverse reaction of R3-1. Swihart and Carr reported that Si_2_Cl_4_ has a monobridged isomer.^32^ However, it is neglected here because it is unstable compared to the other isomers, Cl_3_SiSiCl and Cl_2_SiSiCl_2_ (17.9 kcal/mol relative to Cl_3_SiSiCl), and no reactions whose reactant is the monobridged isomer have been found.

### 3.5 Thermodynamics Parameters

Some calculated thermodynamic parameters of the reactions are compared to available experimental data in Table 2. The calculated thermodynamic parameters are in good agreement with the experimental data.^33,34^ The maximum differences between the calculated enthalpies and entropies of reaction and available experimental data are 1.3 kcal/mol and 1.7 cal/mol/K, respectively.

**Table 2:** Comparison of Predicted Enthalpies and Entropies of Reaction with Experimental Data

		Δ*H*^0^(kcal mol^–1^)		Δ*S*^*0*^(cal K^–1^ mol^–1^)	
reaction		calculated	experimental	calculated	experimental
R1-1	Cl_3_SiSiH_2_Cl → SiH_2_Cl_2_ + SiCl_2_	33.1	32.8^a^^b^	35.9	36.5^a^
R1-2	Cl_3_SiSiH_2_Cl → SiHCl_3_ + SiHCl	44.0	44.8^a^	35.1	35.3^a^
R1-4	Cl_3_SiSiH_2_Cl → SiCl_4_ + SiH_2_	53.1	53.1^a^	28.7	29.0^a^
R1-7	HCl_2_SiSiHCl_2_ → SiHCl_3_ + SiHCl	43.5	44.8^a^	36.2	34.5^a^
R1-8	HCl_2_SiSiHCl_2_ → SiH_2_Cl_2_ + SiCl_2_	32.5	32.8^a^^b^	37.1	35.7^a^
R2-1	Si_2_Cl_6_ → SiCl_4_ + SiCl_2_	34.5	34.6^a^^b^	36.1	35.5^a^
R2-2	Si_2_Cl_6_ → SiCl_3_ + SiCl_3_	78.1	77.4^a^	42.5	41.3^a^

a Chase.^33^

b Walker et al.^34^

### 3.6 Hindered Rotor Analysis

**Figure 5:**
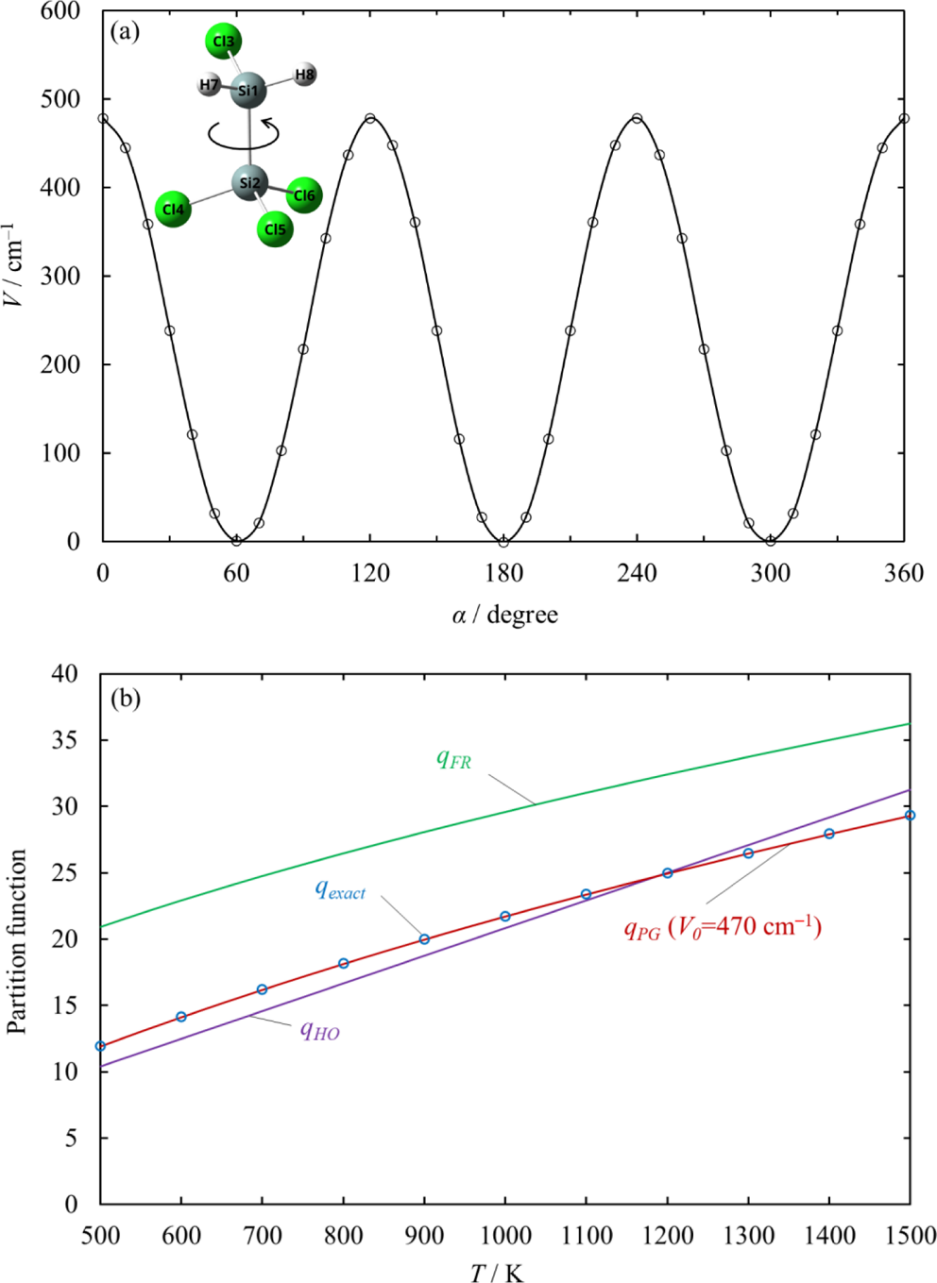
Hindered rotor analysis for the SiH_2_Cl rotor in Cl_3_SiSiH_2_Cl: (a) potential energy curve torsional angle α is defined as the dihedral angle between atoms Cl3–Si1–Si2–Cl5. The solid curve represents the Fourier series interpolation. (b) Partition function calculated from eigenstate energies (*q*_exact_) in comparison with the harmonic oscillator (*q*_HO_), free rotor (*q*_FR_), and Pitzer–Gwinn (*q*_PG_) approximations.

Figure 5 shows the hindered rotor analysis for the SiH_2_Cl rotor in Cl_3_SiSiH_2_Cl as an example. All the partition functions in Figure 5b were calculated consistently with the energy relative to the bottom of the potential energy curve in Figure 5a in a wide range of temperature. The partition function calculated via the Pitzer–Gwinn approximation with a hindrance potential height of *V*_0_ = 470 cm^–1^ shows extremely good agreement with the partition function calculated from the eigenstate energies by BEx1D (*q*_exact_) in a wide range of temperatures. Therefore, this internal rotation is treated as hindered with the hindrance potential height of 470 cm^–1^.

Similarly, the results of the hindered rotor analyses for other species and tight TSs that have torsional motion at the Si–Si bond are shown in the Supporting Information (S3). These tight TSs have the torsional motion at the Si–Si bond because no atom moves between the two Si atoms during the elimination of H_2_ or HCl. The estimated barrier heights of Pitzer–Gwinn approximation *V*_0_ and the symmetry number of the internal rotation σ_int_ are listed in Table 3.

**Table 3:** Barrier Heights for Hindered Rotations in eq 1

species	*V*_0_/cm^–1^	σ_int_
Cl_3_SiSiH_2_Cl	470	3
HCl_2_SiSiHCl_2_	660	1
Si_2_Cl_6_	640	3
Cl_3_SiSiCl	40	3
TS4 (Cl_3_SiSiH_2_Cl → Cl_3_SiSiCl + H_2_)	890	3
TS6 (Cl_3_SiSiH_2_Cl → Cl_3_SiSiH + HCl)	590	3
TS11 (HCl_2_SiSiHCl_2_ → HCl_2_SiSiCl + HCl)	790	1

**Figure 6:**
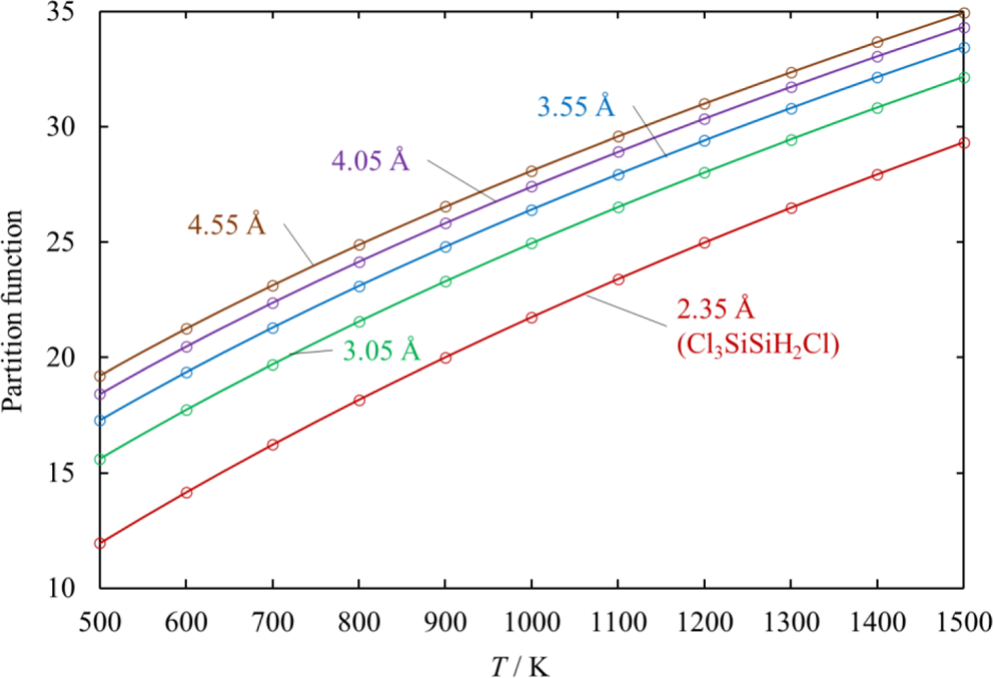
Hindered rotor partition function *q*(*r*) in eq r2 at *r* = 2.35 (Cl_3_SiSiH_2_Cl), 3.05, 3.55, 4.05, and 4.55 Å for R1-6.

**Figure 7:**
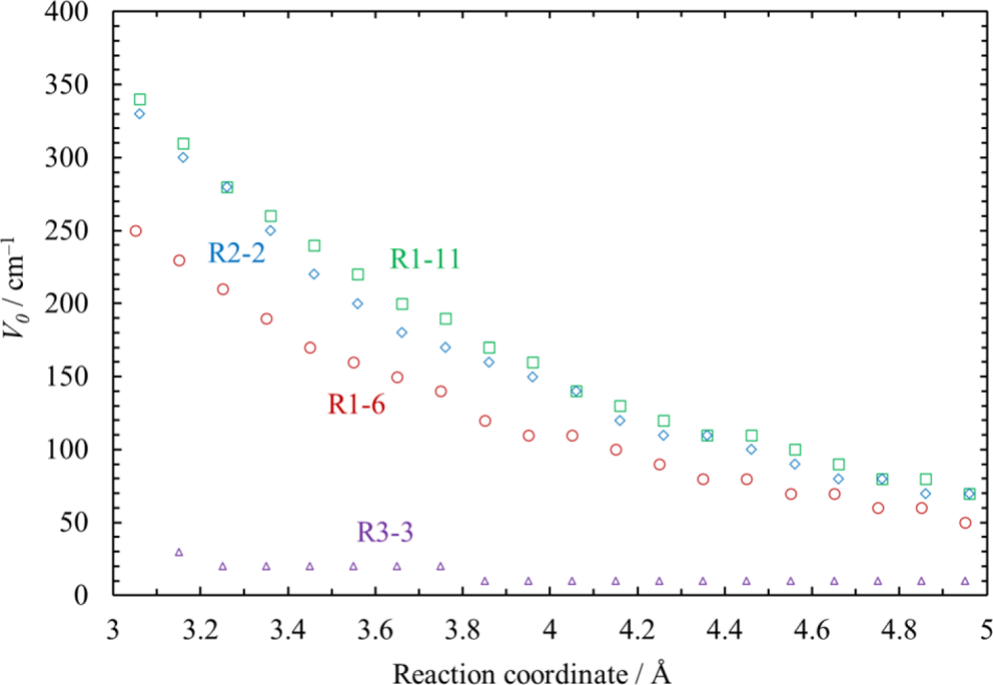
Estimated values of hindered barrier heights *V*_0_ for each reaction coordinate and for the simple bond fission reactions R1-6, R1-11, R2-2, and R3-3.

For the simple Si–Si bond fission reactions R1-6, R1-11, R2-2, and R3-3, the hindered rotor partition functions for each reaction coordinate are calculated using eq 2. The partition function *q*(*r*) in eq 2 for some values of the reaction coordinate *r* of R1-6 is shown in Figure 6 as an example. The hindered barrier height *V*_0_ of Pitzer–Gwinn approximation eq 1 for each reaction coordinate was estimated so that *q*_PG_ agrees with the corresponding *q*(*r*). Estimated values of *V*_0_ for each reaction are plotted in Figure 7. As the Si–Si bond stretches, the hindered rotor partition function increases and the hindered rotor approaches a free rotor.

**Figure 8:**
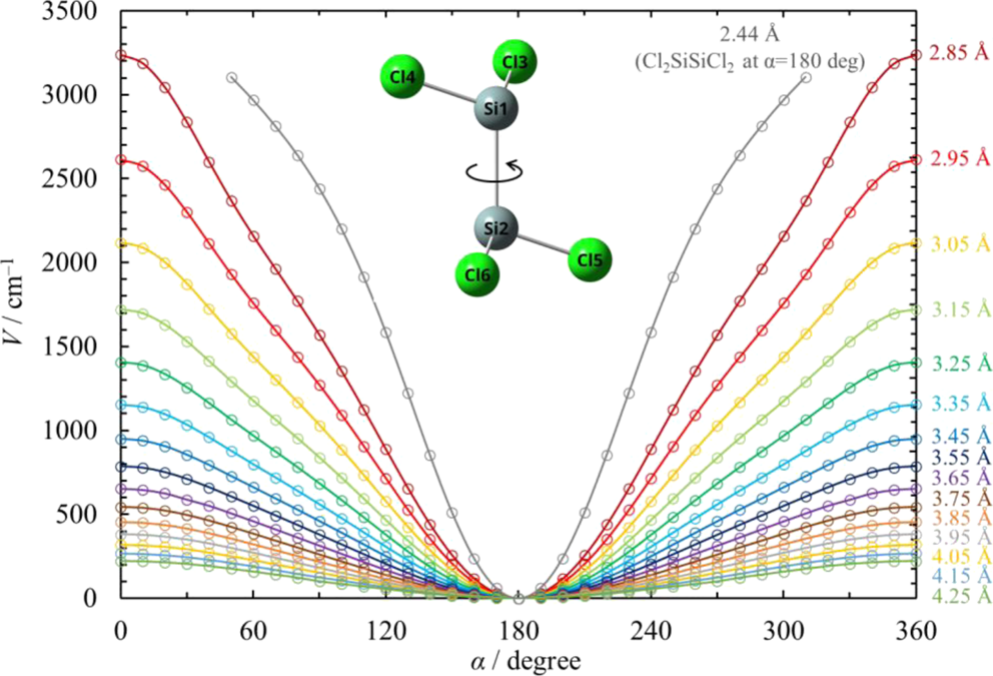
Potential energy curve for torsional motion at the Si–Si bond of Cl_2_SiSiCl_2_ at various values of the reaction coordinate of R3-2. Torsional angle α is defined as the dihedral angle between atoms Cl3–Si1–Si2–Cl6. The solid curve represents the Fourier series interpolation.

Cl_2_SiSiCl_2_ and its simple bond fission reaction R3-2 are extraordinary cases in this work. Cl_2_SiSiCl_2_ is not planar but a trans-bent structure, as shown in Figure 8. Therefore, it becomes unstable when each Cl atom in the SiCl_2_ moieties comes close during the torsional motion of the Si–Si bond. At that moment, Cl_2_SiSiCl_2_ can become a more stable isomer Cl_3_SiSiCl, that is, isomerization −R3-1 can occur. This behavior is similar to that of Si_2_H_4_.^35^ The potential energy curves for the torsional motion of Cl_2_SiSiCl_2_ and various points of the reaction coordinate of R3-2 are depicted in Figure 8.

The potential energy curve for torsional motion at the Si–Si bond of Cl_2_SiSiCl_2_ was calculated. The torsional angle α is defined as the dihedral angle between atoms Cl3–Si1–Si2–Cl6 here. The potential energies at the reaction coordinate *r* < 2.85 Å and α ≤ 50° or α ≥ 310° are not shown in Figure 8 because the optimization converged to the structure of Cl_3_SiSiCl.

**Figure 9:**
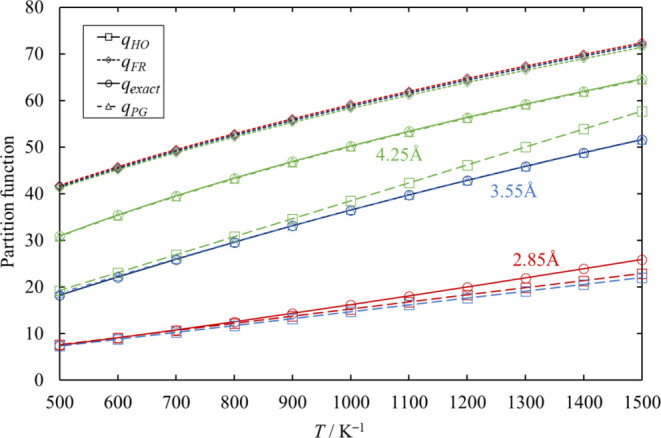
Partition functions calculated from eigenstate energies (*q*_exact_) in comparison with the harmonic oscillator (*q*_HO_), free rotor (*q*_FR_), and Pitzer–Gwinn (*q*_PG_) approximations at reaction coordinate *r* = 2.85, 3.55, and 4.25 Å as representative values. *q*_PG_ at *r* = 2.85 Å is not shown. At *r* = 3.55 and 4.25 Å, the values of *V*_0_ in *q*_PG_ are 770 and 220 cm^–1^, respectively.

**Figure 10:**
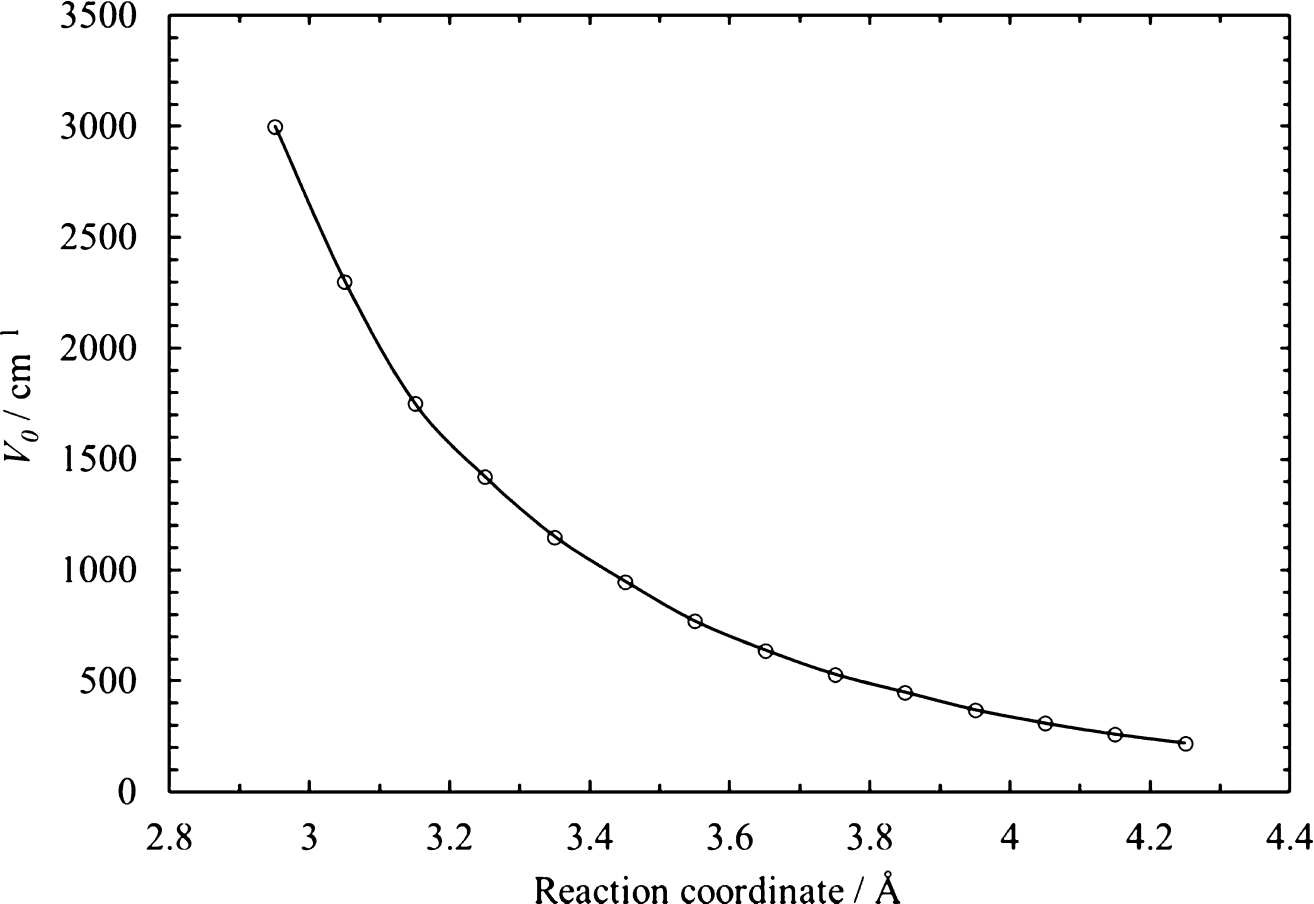
Estimated values of hindered barrier heights *V*_0_ for each reaction coordinate and for the simple bond fission reactions R3-2.

The partition function calculated from eigenstate energies (*q*_exact_) are shown in Figure 9 in comparison with that of a harmonic oscillator (*q*_HO_), of a free rotor (*q*_FR_), and of Pitzer–Gwinn (*q*_PG_) approximations at values of reaction coordinate *r* = 2.85, 3.55, and 4.25 Å as representative values. When *r* ≤ 2.85 Å, Pitzer–Gwinn approximation could not reproduce the values of *q*_exact_, and the harmonic oscillator is a fairly good assumption. Therefore, for Cl_2_SiSiCl_2_ and reaction coordinate *r* ≤ 2.85 Å in R3-2, the torsional motions at the Si–Si bond were assumed to be harmonic oscillations, and at *r* ≥ 2.85 Å in R3-2, the torsional motions at the Si–Si bond were treated as Pitzer–Gwinn rotors. The estimated values of *V*_0_ at reaction coordinate *r* ≥ 2.85 Å for R3-2 are plotted in Figure 10.

### 3.7 Rate Coefficient Calculation

#### 3.7.1 Unimolecular Decomposition of Si_2_H_2_Cl_4_

**Figure 11:**
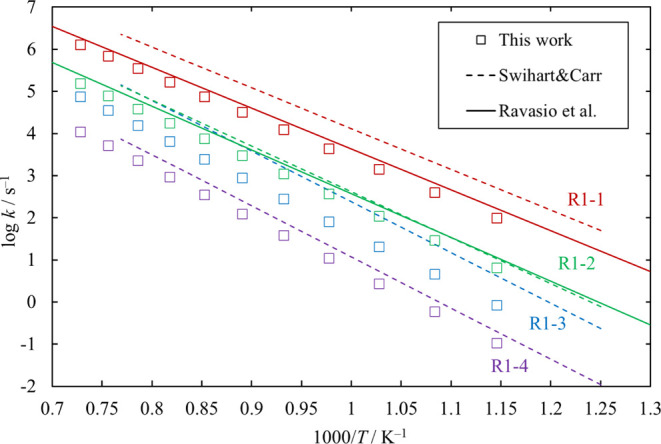
High-pressure-limit rate coefficients of the unimolecular dissociation channels of Cl_3_SiSiH_2_Cl. Rate coefficients reported in the literature (Ravasio et al.^3^ for R1-1 and R1-2 and Swihart and Carr^4^ for R1-1, R1-2, R1-3, and R1-4) are also shown for comparison.

The high-pressure-limit rate coefficients of all dissociation channels investigated in this work were calculated through the TST using the GPOP^18^ program suite. The results for R1-1, R1-2, R1-3, and R1-4 are plotted in Figure 11 together with some data published in the literature. The rate coefficients obtained in this work are in good agreement with the values of Ravasio et al.^3^ for R1-1 and R1-2 and a little smaller than the values of Swihart and Carr^4^ for R1-1, R1-2, R1-3, and R1-4.

**Figure 12:**
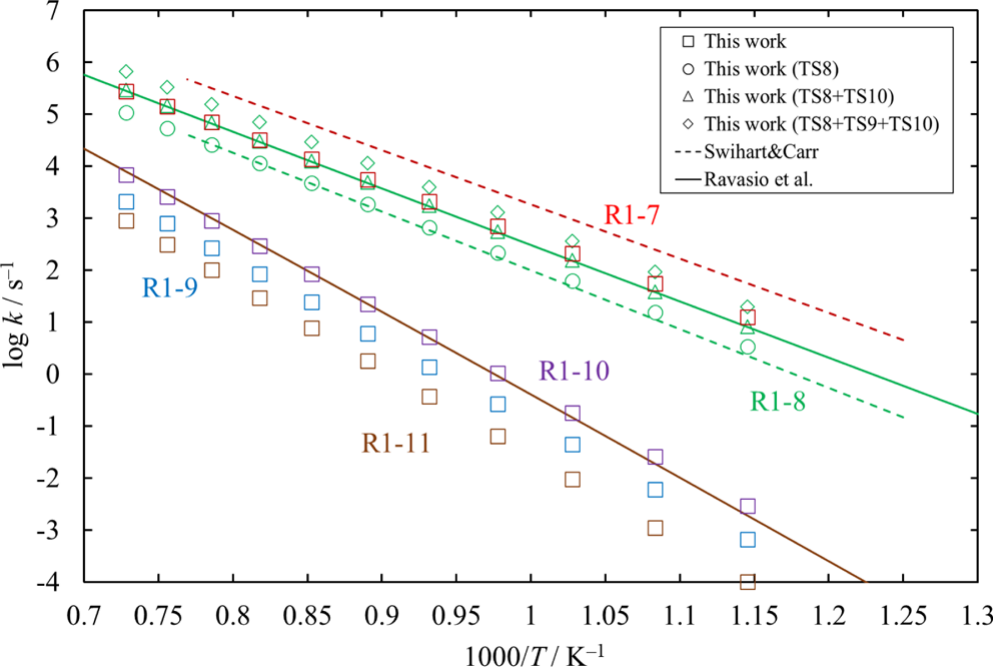
High-pressure-limit rate coefficients of the unimolecular dissociation channels of HCl_2_SiSiHCl_2_, R1-7, R1-8, R1-9, R1-10, and R1-11. Rate coefficients reported in the literature (Ravasio et al.^3^ and Swihart and Carr^4^) are also shown, for comparison.

The high-pressure-limit rate coefficients of all dissociation channels in the HCl_2_SiSiHCl_2_ potential well were calculated. The results for R1-7, R1-9, and R1-11 are plotted in Figure 12 together with some data published in the literature.^3,4^ The results for R1-10 are also plotted in the figure, but there are no literature data for R1-10. The rate coefficients obtained in this work for R1-7 are a little smaller than the values reported in the literature.^4^ As shown in Figure 2, we found three TSs for R1-8 (TS8, TS9, and TS10). Swihart and Carr reported only TS8 as the TS for R1-8. Ravasio et al. reported TS8 and TS10 and gave the total reaction rate coefficient as the sum of the rate coefficients related to the two channels. In this work, the total reaction rate coefficient of R1-8 is calculated as the sum of the rate coefficients related to TS8, TS9, and TS10, but the rate coefficients of R1-8 are shown in the figure for three different combinations, for comparison. The rate coefficients related to TS8 are in good agreement with the value shown by Swihart and Carr. The sum of the rate coefficients related to TS8 and TS10 agrees with the value shown by Ravasio et al.

Next, we estimated the pressure dependence of each of the unimolecular decomposition channels of Cl_3_SiSiH_2_Cl from R1-1 to R1-6 and HCl_2_SiSiHCl_2_ from R1-7 to R1-10. Channels R1-6 and R1-10 proceed through loose TSs, and their rate coefficients at various values of pressure were determined through the microcanonical variational TST (MVTST) and using SSUMES.^22^

**Figure 13:**
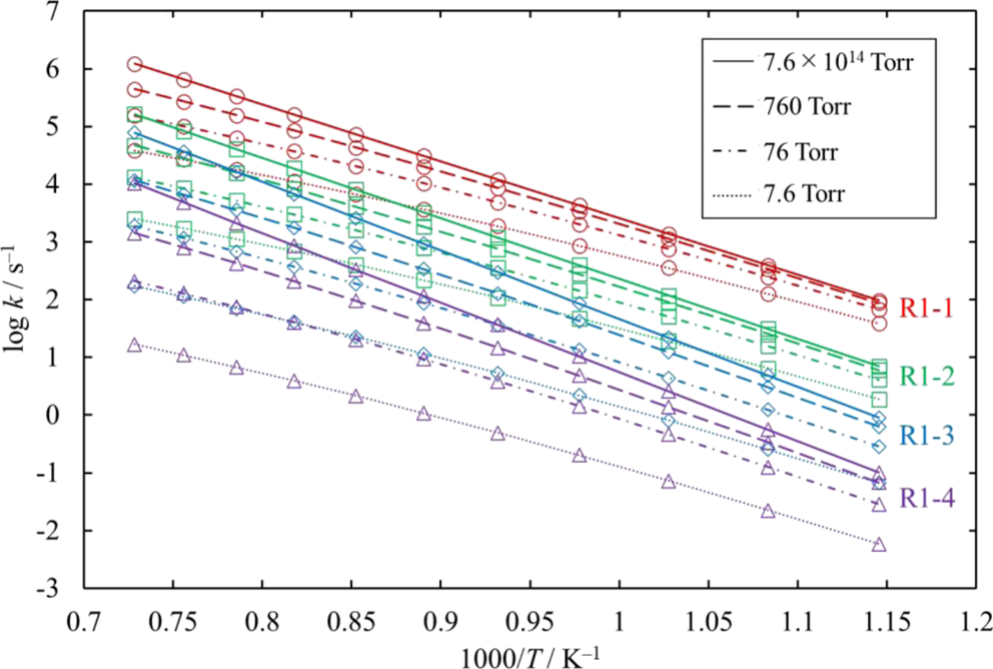
Rate coefficients calculated for the unimolecular dissociation channels of Cl_3_SiSiH_2_Cl under various conditions of temperature and pressure. The rate coefficients for R1-5 and R1-6 are small and therefore omitted. The lines pass through all the calculated values (symbols).

**Figure 14:**
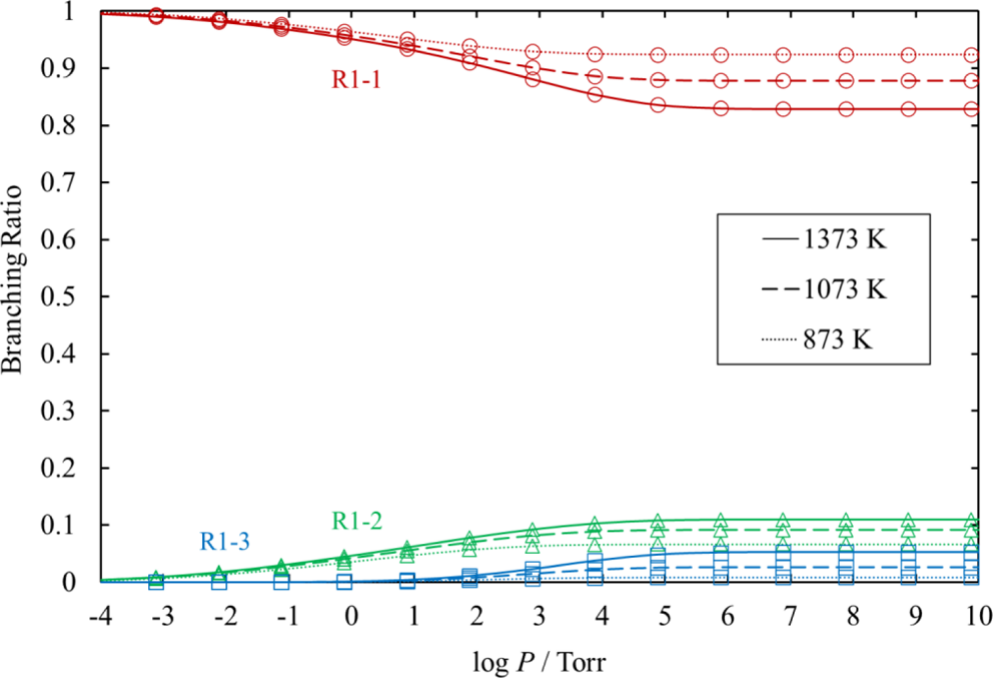
Branching ratios for the unimolecular dissociation channels of Cl_3_SiSiH_2_Cl under various conditions of temperature and pressure. Ratios for R1-4, R1-5, and R1-6 are small and therefore omitted. The lines pass through all the calculated values (symbols).

**Figure 15:**
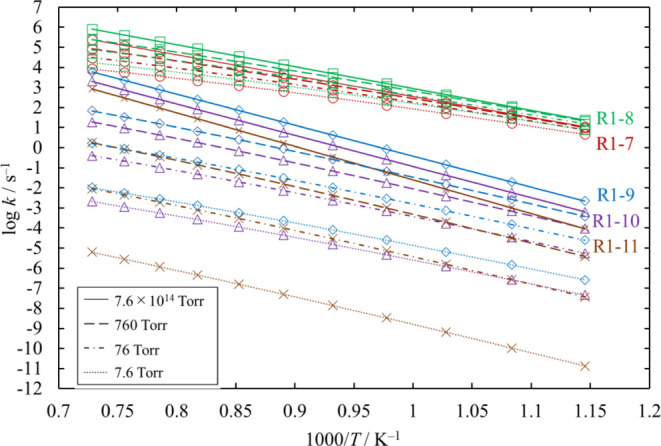
Rate coefficients calculated for the unimolecular dissociation channels of HCl_2_SiSiHCl_2_ under various conditions of temperature and pressure. The lines pass through all the calculated values (symbols).

**Figure 16:**
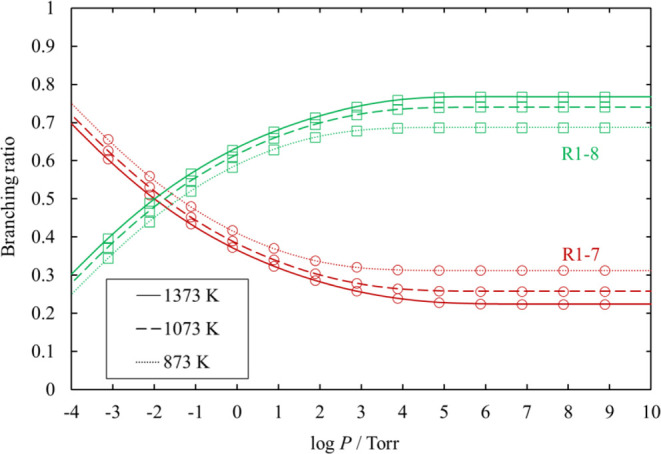
Branching ratios for the unimolecular dissociation channels of HCl_2_SiSiHCl_2_ under various conditions of temperature and pressure. Ratios for R1-9, R1-10, and R1-11 are small and therefore omitted. The lines pass through all the calculated values (symbols).

The rate coefficients and the branching ratios related to the Cl_3_SiSiH_2_Cl and HCl_2_SiSiHCl_2_ potential wells, R1-1–R1-4 for various values of temperature and pressure are plotted in Figures 13–16. The results of the reactions whose branching ratios were always smaller than 0.01 were omitted for clarity. For the dissociation reactions in the Cl_3_SiSiH_2_Cl potential well, the dominant channel is R1-1, with branching ratios always larger than 0.83. The branching ratios of R1-2 and R1-3 are at most 0.11 and 0.05, respectively. For the dissociation reactions in the HCl_2_SiSiHCl_2_ potential well, the activation energy of R1-7 is lower than that of R1-8. However, the total rate coefficient of R1-8 is larger than that of R1-7, except under very low pressure, because R1-8 proceeds through three TSs.

#### 3.7.2 Chemical Activation SiH_2_Cl_2_ + SiCl_2_ Reaction

As reverse channels to the unimolecular decomposition of Si_2_H_2_Cl_4_, the channels that start from the products of R1-1–R1-11 could proceed as chemical activation reactions. However, only SiH_2_Cl_2_ + SiCl_2_ and SiHCl_3_ + SiHCl are expected to proceed at significant rates if we assume CVD conditions where DCS or TCS is used as the silicon source.^3,4^ Therefore, the other chemical activation channels will not be discussed here. The possible products of the chemical activation reaction SiH_2_Cl_2_ + SiCl_2_ are:
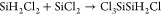
–R1-1
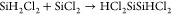
–R1-8
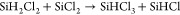
R1-12
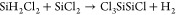
R1-13
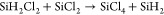
R1-14
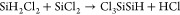
R1-15
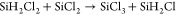
R1-16
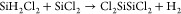
R1-17
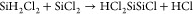
R1-18
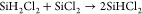
R1-19

R1-12 can proceed through TS1, or TS2, and TS3 in the direction of the Cl_3_SiSiH_2_Cl well (left side of Figure 2) or through TS8, TS9, or TS10, and TS7 in the direction of the HCl_2_SiSiHCl_2_ well (right side of Figure 2). In this work, the rate coefficient of R1-12 is calculated as the sum of the rate coefficients of these five reaction paths.

**Figure 17:**
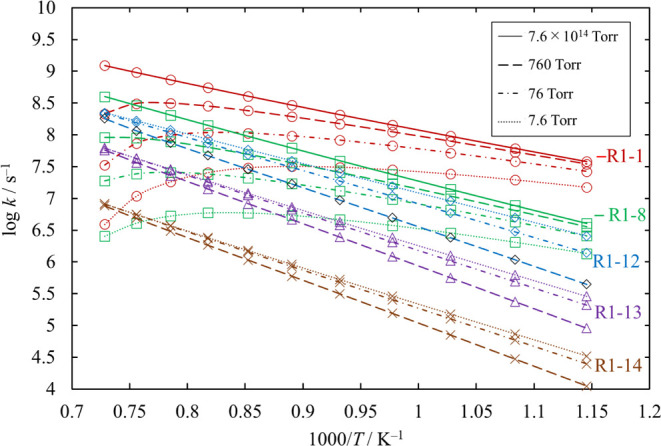
Rate coefficients of the chemical activation channels proceeding from SiH_2_Cl_2_ + SiCl_2_, as functions of temperature, at various values of pressure. The results at 7.6 × 10^14^ Torr are only plotted for the recombination reactions –R1-1 and –R1-8. The rate coefficients for R1-15–R1-19 are small and therefore omitted. The lines pass through all the calculated values (symbols).

**Figure 18:**
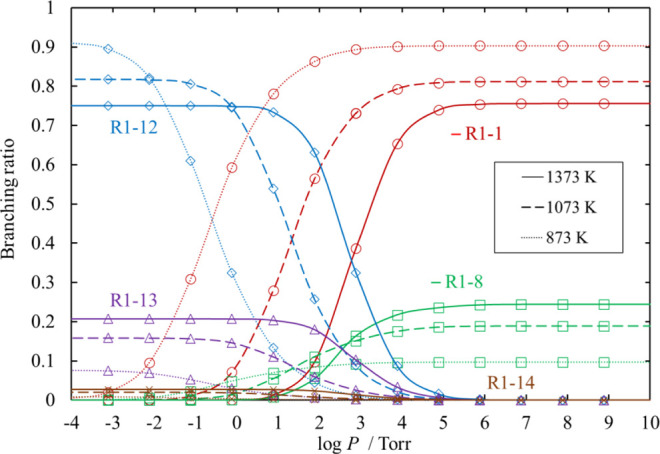
Branching ratios for the chemical activation channels of SiH_2_Cl_2_ + SiCl_2_ under various conditions of temperature and pressure. Ratios for R1-15–R1-19 are small and therefore omitted. The lines pass through all the calculated values (symbols).

The rate coefficients and the branching ratios of the chemical activation reaction SiH_2_Cl_2_ + SiCl_2_ for various values of temperature and pressure are plotted in Figures 17 and 18, respectively. The recombination reactions –R1-1 and –R1-8 are the dominant channels under high pressure. Under moderate or lower pressure, R1-12 and R1-13 compete with –R1-1 and –R1-8, and the branching ratio of even R1-14 exceeds 0.01.

#### 3.7.3 Chemical Activation SiHCl_3_ + SiHCl Reaction

The possible chemical activation reactions for SiHCl_3_ + SiHCl are:
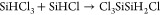
–R1-2
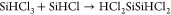
–R1-7
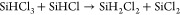
–R1-12
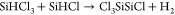
R1-20
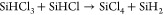
R1-21
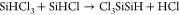
R1-22
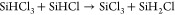
R1-23
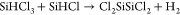
R1-24
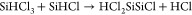
R1-25
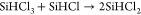
R1-26

**Figure 19:**
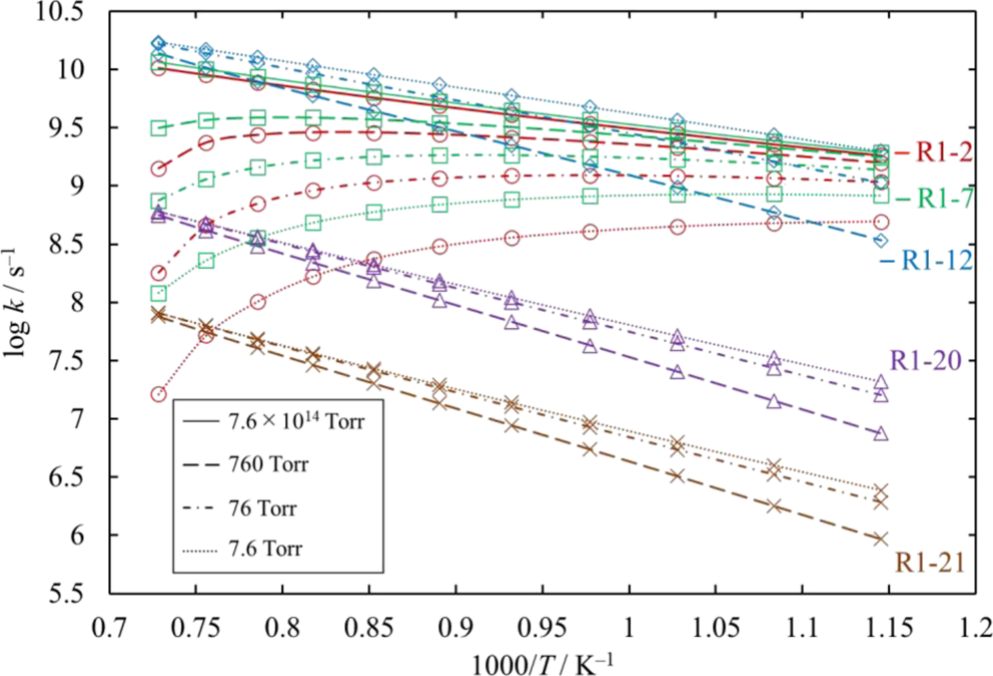
Rate coefficients of the chemical activation channels proceeding from SiHCl_3_ + SiHCl, as functions of temperature, at various values of pressure. The results at 7.6 × 10^14^ Torr are only plotted for the recombination reactions –R1-2 and –R1-7. The rate coefficients for R1-22–R1-26 are small and therefore omitted. The lines pass through all the calculated values (symbols).

**Figure 20:**
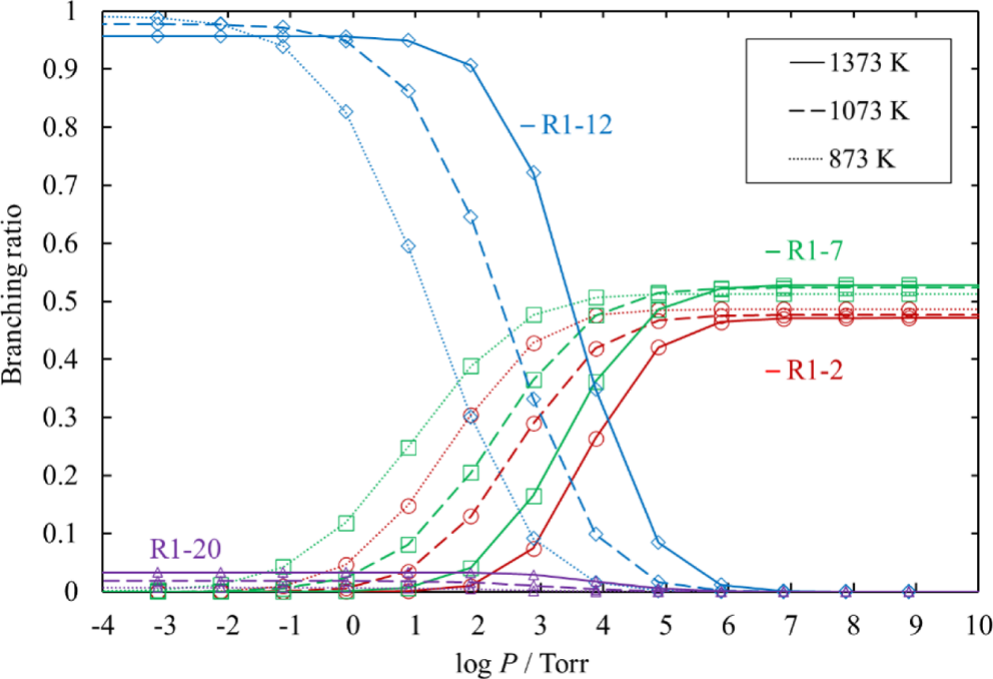
Branching ratios for the chemical activation channels of SiHCl_3_ + SiHCl under various conditions of temperature and pressure. Ratios for R1-21–R1-26 are small and therefore omitted. The lines pass through all the calculated values (symbols).

Similarly to the case of R1-12, the total rate coefficient for –R1-12 is calculated as the sum of the rate coefficients of each possible reaction path. As plotted in Figures 19 and 20, both recombination reactions, –R1-2 and –R1-7, are the dominant channels at high pressure. Under moderate or lower pressure, –R1-12 competes with –R1-2 and –R1-7; the branching ratio of even R1-20 exceeds 0.01. The rate coefficient of the chemical activation R1-24 was small enough to be neglected when compared with that of bimolecular reaction r6 in Ravasio et al.’s work.^3^

### 3.8 Reactions Related to the Si_2_Cl_6_ Potential Well

#### 3.8.1 Unimolecular Decomposition of Si_2_Cl_6_

**Figure 21:**
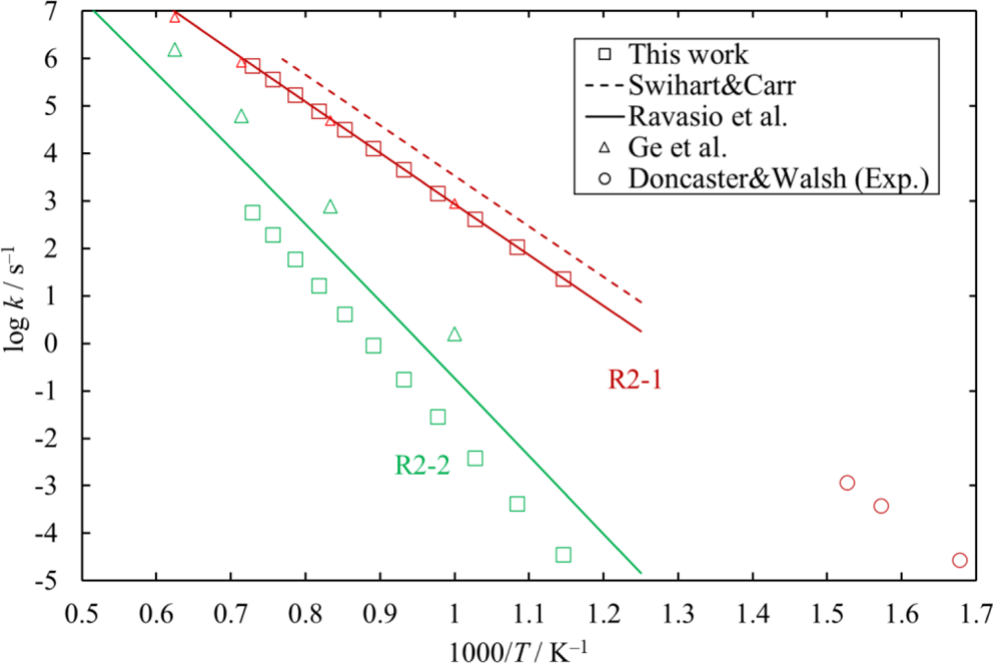
High-pressure-limit rate coefficients of the unimolecular dissociation channels of Si_2_Cl_6_. Rate coefficients reported in the literature (Ravasio et al.^3^ for R2-1 and R2-2; Swihart and Carr^4^ for R2-1; Ge et al.^15^ for R2-1 and R2-2; and Doncaster and Walsh^17^ for R2-1) are also shown, for comparison.

The high-pressure-limit rate coefficients of all Si_2_Cl_6_ dissociation channels investigated in this work were calculated. The results for R2-1 and R2-2 are plotted in Figure 21 together with some data published in the literature. The results for R2-1 are in good agreement with the calculated values by Ravasio et al.^3^ and Ge et al.^15^ and experimental values by Doncaster and Walsh.^17^ Doncaster and Walsh obtained the rate coefficients at 10 Torr and temperatures within the range 596–655 K. They suggested that the reaction was in its high pressure limit. The results for R2-2 obtained in this work are smaller than the values reported by Ravasio et al.^3^ and Ge et al.^15^

**Figure 22:**
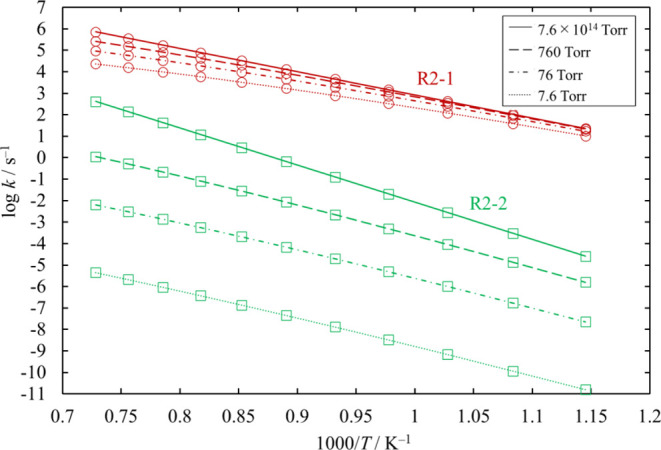
Rate coefficients calculated for the unimolecular dissociation channels of Si_2_Cl_6_ under various conditions of temperature and pressure. The lines pass through all the calculated values (symbols).

We estimated the pressure dependence of each of the unimolecular decomposition channels of Si_2_Cl_6_, R2-1 and R2-2. The results are plotted in Figure 22. Channel R2-2 proceeds through loose TSs, and their rate coefficients at various values of pressure were determined through the MVTST. The dominant channel is R2-1, and the rate coefficients for R2-2 are small enough to be neglected under all pressure conditions.

#### 3.8.2 Chemical Activation SiCl_3_ + SiCl_3_ Reaction

According to the PSR simulation of Ravasio et al.,^3^ which had H_2_ and SiHCl_3_ as the feed gas, SiCl_3_ is the most abundant radical, and the recombination reaction –R2-2 is in general the fastest.
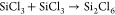
–R2-2
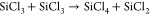
R2-3

**Figure 23:**
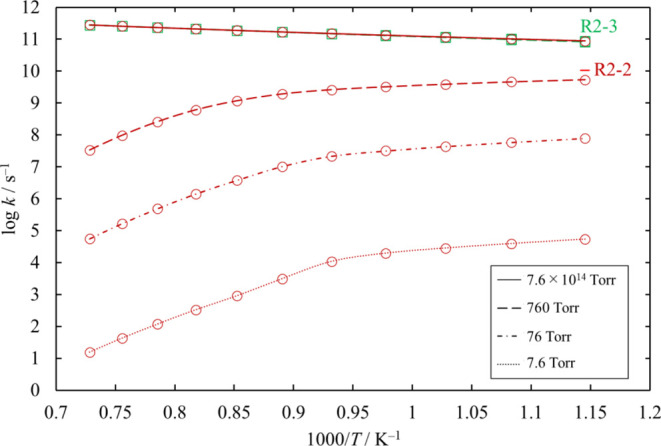
Rate coefficients of the chemical activation channels proceeding from SiCl_3_ + SiCl_3_, as functions of temperature, at various values of pressure. The results at 7.6 × 10^14^ Torr are only shown for the recombination reaction –R2-2. The plots of R2-3 at 760, 76, and 7.6 Torr overlap. The lines pass through all the calculated values (symbols).

**Figure 24:**
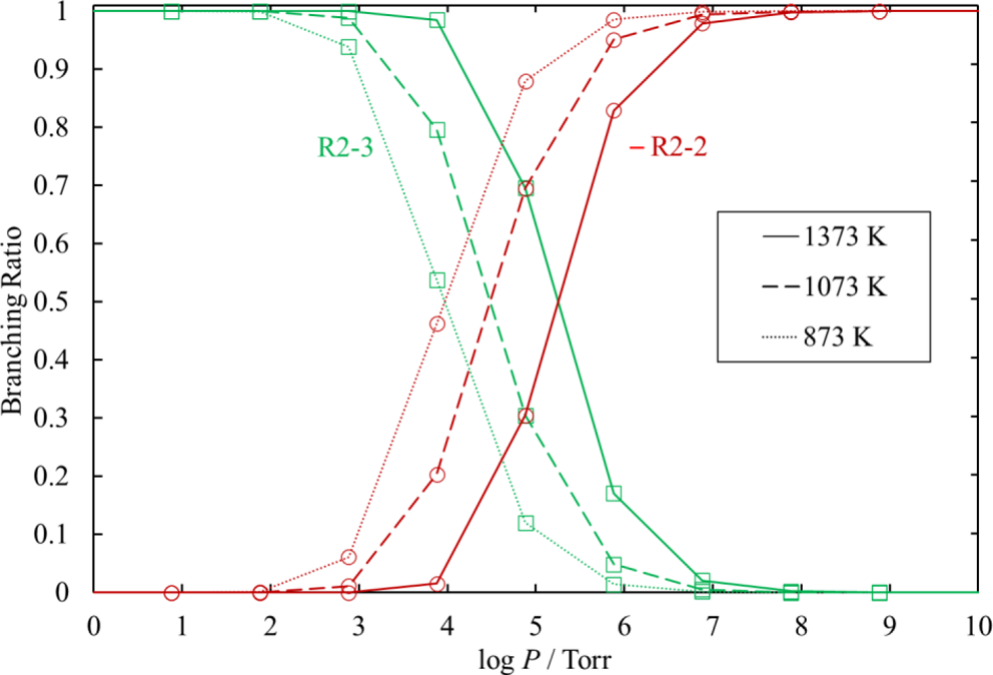
Branching ratios for the chemical activation channels of SiCl_3_ + SiCl_3_ under various conditions of temperature and pressure. The lines pass through all the calculated values (symbols).

The rate coefficients and branching ratios of these reactions for various values of temperature and pressure are plotted in Figures 23 and 24, respectively. At moderate or lower pressure, the chemical activation reaction R2-3 is the dominant channel, and it no longer shows pressure dependence.

### 3.9 Reactions Related to the Si_2_Cl_4_ Potential Well

#### 3.9.1. Unimolecular Decompsition and Isomerization of Si_2_Cl_4_

**Figure 25:**
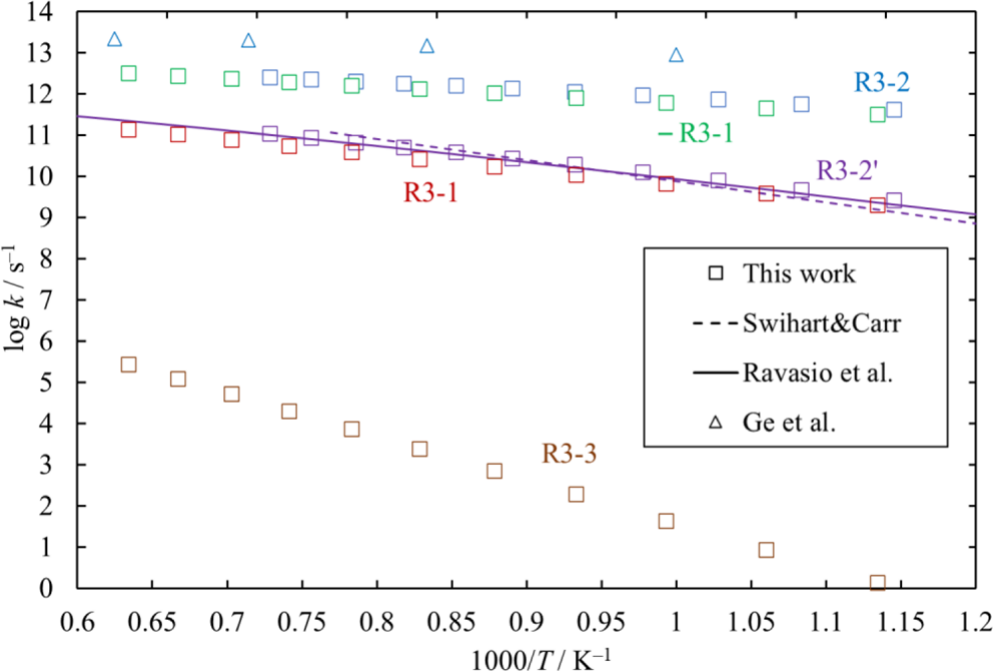
High-pressure-limit rate coefficients of the unimolecular isomerization and dissociation channels of Si_2_Cl_4_. Rate coefficients reported in the literature (Ravasio et al.^3^ for R3-2′; Swihart and Carr^4^ for R3-2′; and Ge et al.^15^ for R3-2) are also shown, for comparison.

The rate coefficients for in high pressure limit are shown in Figure 25 for comparison with the literature values.

**Figure 26:**
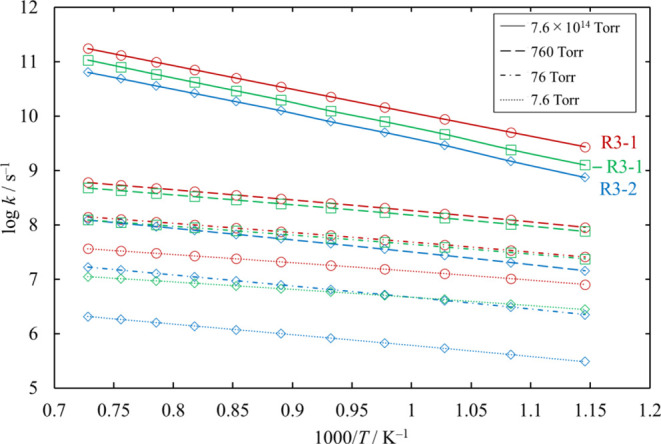
Rate coefficients of the unimolecular dissociation channels of Si_2_Cl_4_, as functions of temperature, at various values of pressure. The rate coefficients for R3-3 are small and therefore omitted. The lines pass through all the calculated values (symbols).

We estimated the pressure dependence of each of the unimolecular decomposition channels of Si_2_Cl_4_, R3-1, R3-2, and R3-3. Channels R3-2 and R3-3 proceed through loose TSs, and their rate coefficients at various values of pressure were determined through the MVTST. Note that the rate coefficients for R3-2 at 7.6 × 10^14^ Torr in Figure 26 reach the high pressure limit, but it is smaller than the values in Figure 25, which are calculated through TST.

Since the activation energy of R3-3 is much higher than that of R3-1, the rate coefficient and the branching ratio in the Cl_3_SiSiCl potential energy well of R3-3 are small enough to be neglected, and almost all reactive Cl_3_SiSiCl change to Cl_2_SiSiCl_2_ through R3-1.

**Figure 27:**
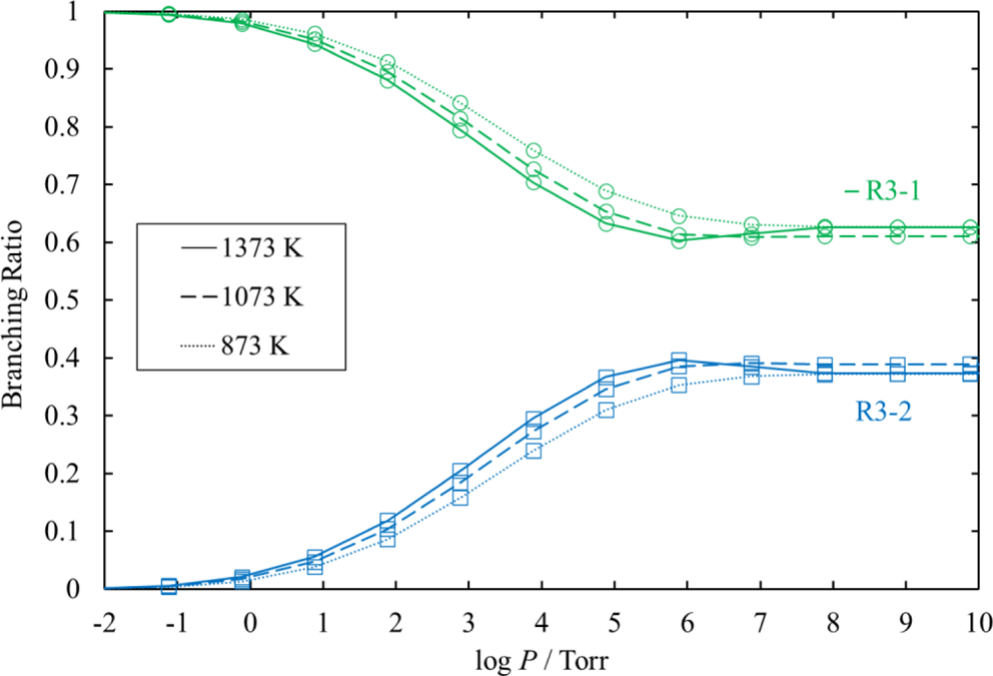
Branching ratios for the unimolecular dissociation channels of Cl_2_SiSiCl_2_ under various conditions of temperature and pressure. The lines pass through all the calculated values (symbols).

The branching ratios of reactions for Cl_2_SiSiCl_2_, −R3-1 and R3-2, are similar, and they can compete at moderate or higher pressure, as shown in Figure 27.

#### 3.9.2 Chemical Activation SiCl_3_ + SiCl Reaction

The chemical activation SiCl_3_ + SiCl reactions are:
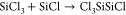
–R3-3
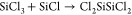
R3-4
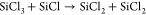
R3-5

**Figure 28:**
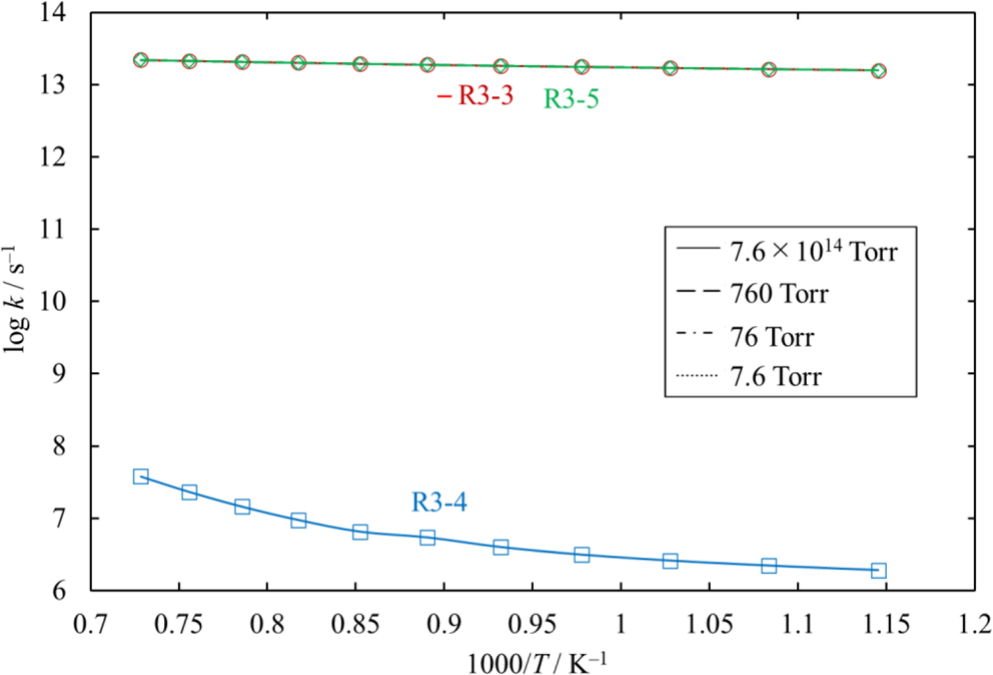
Rate coefficients of the chemical activation channels proceeding from SiCl_3_ + SiCl, as functions of temperature, at various values of pressure. The results at 7.6 × 10^14^ Torr are only shown for the recombination reaction –R3-3. The lines pass through all the calculated values (symbols). The values for –R3-3 at 7.6 × 10^14^ Torr and R3-5 at 760, 76, and 7.6 Torr overlap. The values for R3-4 at each pressure also overlap.

**Figure 29:**
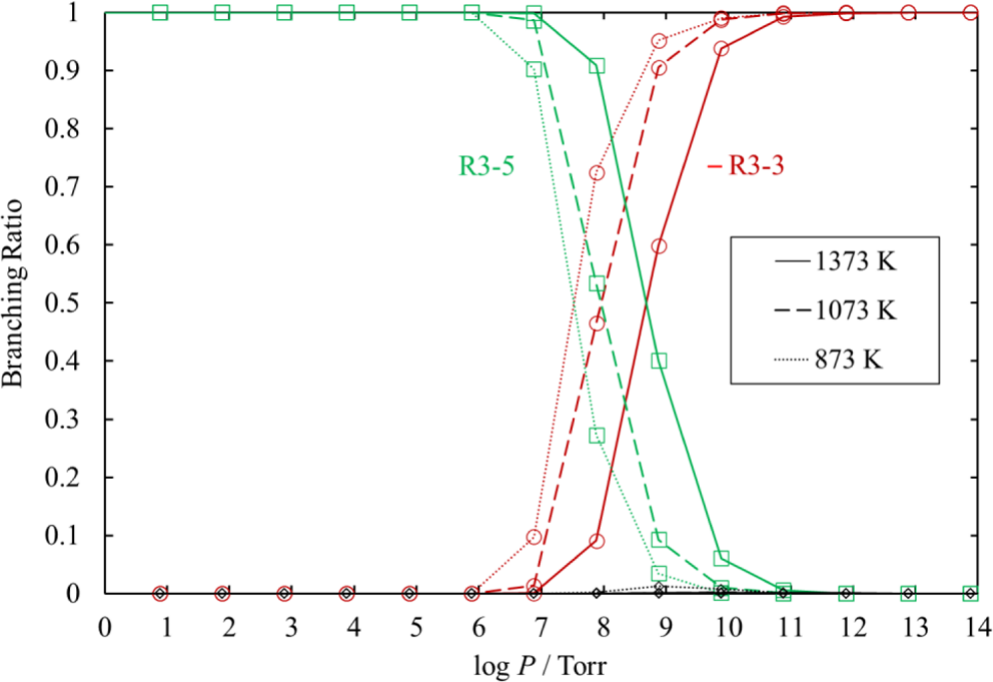
Branching ratios for the chemical activation channels of SiCl_3_ + SiCl under various conditions of temperature and pressure. The branching ratio for R3-4 is at most 0.13% at 873 K and 7.6 × 10^8^ Torr. The lines pass through all the calculated values (symbols).

As shown in Figures 28 and 29, R3-5 is the dominant channel at moderate or lower pressure. Therefore, Si_2_Cl_4_ is hardly produced from the reactions of SiCl_3_ and SiCl at such pressure. This is caused by the high energy of SiCl_3_ + SiCl in the Si_2_Cl_4_ potential energy well. Instead, Si_2_Cl_4_ can be produced through the reaction R1-13 in the Si_2_H_2_Cl_4_ potential well.

### 3.10 Parameterization of Rate Coefficients

The rate coefficients of the channels that seem to be important were parameterized in Table 4 using the modified Arrhenius expression, which is defined as:
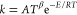
4

**Table 4:** Parameters in the Modified Arrhenius Expression eq 4 in Units Consistent with Centimeters, Seconds, and Moles^a^

no	reactions	pressure	*A*	β	*E* (kcal/mol)	max relative error (%)
R1-1	Cl_3_SiSiH_2_Cl →SiH_2_Cl_2_ + SiCl_2_	HPL	1.00 × 10^11^	0.646	43.6	0.128
		10 atm	1.86 × 10^26^	–3.85	51.8	1.12
		1 atm	1.72 × 10^42^	–8.65	59.4	1.27
		0.1 atm	3.43 × 10^53^	–12.2	63.2	0.795
R1-2	Cl_3_SiSiH_2_Cl → SiHCl_3_ + SiHCl	HPL	3.00 × 10^10^	0.677	46.4	0.126
		10 atm	2.10 × 10^28^	–4.59	55.9	1.16
		1 atm	3.62 × 10^44^	–9.50	63.2	1.20
		0.1 atm	5.66 × 10^54^	–12.8	66.0	0.676
R1-3	Cl_3_SiSiH_2_Cl → Cl_3_SiSiCl + H_2_	HPL	1.56 × 10^11^	0.667	52.7	0.190
		10 atm	2.26 × 10^34^	–6.21	64.6	1.23
		1 atm	5.17 × 10^49^	–11.0	70.5	1.03
		0.1 atm	8.83 × 10^56^	–13.5	71.0	0.499
R1-7	HCl_2_SiSiHCl_2_ → SiHCl_3_ + SiHCl	HPL	1.33 × 10^11^	0.524	46.4	0.122
		10 atm	4.02 × 10^27^	–4.33	55.2	1.17
		1 atm	1.53 × 10^43^	–9.01	62.6	1.31
		0.1 atm	2.74 × 10^54^	–12.5	66.5	0.920
R1-8	HCl_2_SiSiHCl_2_ →SiH_2_Cl_2_ + SiCl_2_	HPL	4.18 × 10^11^	0.631	48.3	0.127
		10 atm	1.54 × 10^30^	–4.85	58.1	1.21
		1 atm	9.34 × 10^45^	–9.62	65.4	1.26
		0.1 atm	3.07 × 10^56^	–13.0	68.6	0.841
–R1-1	SiH_2_Cl_2_ + SiCl_2_ → Cl_3_SiSiH_2_Cl	HPL	3.85	3.20	9.69	0.0445
		10 atm	2.15 × 10^28^	–4.92	25.2	8.66
		1 atm	6.65 × 10^79^	–20.0	53.6	28.6
		0.1 atm	2.02 × 10^146^	–39.6	89.3	49.8
–R1-8	SiH_2_Cl_2_ + SiCl_2_ → HCl_2_SiSiHCl_2_	HPL	8.90	3.20	15.0	0.0453
		10 atm	4.09 × 10^23^	–3.47	27.2	3.18
		1 atm	1.82 × 10^53^	–12.3	42.5	9.21
		0.1 atm	1.55 × 10^93^	–24.2	62.8	19.0
R1-12	SiH_2_Cl_2_ + SiCl_2_ → SiHCl_3_ + SiHCl	10 atm	8.04 × 10^–3^	4.42	23.9	5.59
		1 atm	5.29 × 10^9^	0.874	26.5	3.17
		0.1 atm	2.32 × 10^14^	–0.637	25.3	0.623
R1-13	SiH_2_Cl_2_ + SiCl_2_ → Cl_3_SiSiCl + H_2_	10 atm	7.98 × 10^2^	2.96	29.2	4.61
		1 atm	7.64 × 10^11^	0.211	30.1	2.07
		0.1 atm	8.01 × 10^10^	0.341	26.3	0.410
R1-14	SiH_2_Cl_2_ + SiCl_2_ → SiCl_4_ + SiH_2_	10 atm	5.73 × 10^2^	2.77	30.0	4.29
		1 atm	1.19 × 10^11^	0.211	30.5	1.91
		0.1 atm	2.44 × 10^9^	0.550	26.4	0.403
–R1-2	SiHCl_3_ + SiHCl → Cl_3_SiSiH_2_Cl	HPL	3.76	3.09	1.57	0.00510
		10 atm	9.57 × 10^30^	–5.83	18.4	8.74
		1 atm	9.94 × 10^82^	–21.1	46.7	28.6
		0.1 atm	3.85 × 10^148^	–40.5	81.7	49.7
–R1-7	SiHCl_3_ + SiHCl → HCl_2_SiSiHCl_2_	HPL	13.3	2.96	2.14	0.00711
		10 atm	4.97 × 10^21^	–3.09	13.4	3.17
		1 atm	1.37 × 10^51^	–11.8	28.8	9.30
		0.1 atm	6.26 × 10^91^	–23.9	49.8	19.1
–R1-12	SiHCl_3_ + SiHCl → SiH_2_Cl_2_ + SiCl_2_	10 atm	0.709	3.89	14.0	4.97
		1 atm	1.07 × 10^11^	0.537	16.3	2.96
		0.1 atm	1.45 × 10^15^	–0.826	14.8	0.606
R1-20	SiHCl_3_ + SiHCl →Cl_3_SiSiCl + H_2_	10 atm	2.18 × 10^5^	2.08	20.8	3.35
		1 atm	1.14 × 10^12^	–0.0173	20.5	1.47
		0.1 atm	7.04 × 10^8^	0.768	15.6	0.327
R2-1	Si_2_Cl_6_ → SiCl_4_ + SiCl_2_	HPL	1.03 × 10^12^	0.482	48.2	0.125
		10 atm	5.38 × 10^27^	–4.14	56.6	1.17
		1 atm	3.50 × 10^44^	–9.18	64.7	1.39
		0.1 atm	1.99 × 10^57^	–13.2	69.4	0.985
–R2-2	SiCl_3_ + SiCl_3_ → Si_2_Cl_6_	HPL	1.90 × 10^4^	2.32	0.605	0.0141
		10 atm	1.54 × 10^159^	–43.3	85.2	41.0
		1 atm	8.21 × 10^270^	–76.6	144	29.4
		0.1 atm	(no data)	(no data)	(no data)	(no data)
R2-3	SiCl_3_ + SiCl_3_ → SiCl_4_ + SiCl_2_	10 atm	4.72 × 10^8^	1.18	5.87	0.625
		1 atm	7.61 × 10^6^	1.58	2.43	0.0885
		0.1 atm	9.40 × 10^4^	2.11	0.981	0.376
R3-1	Cl_3_SiSiCl → Cl_2_SiSiCl_2_	HPL	1.28 × 10^13^	0.373	19.0	0.0765
		10 atm	5.59 × 10^19^	–2.49	15.7	0.630
		1 atm	4.92 × 10^16^	–1.86	13.0	0.536
		0.1 atm	5.13 × 10^14^	–1.52	11.3	0.461
–R3-1	Cl_2_SiSiCl_2_ → Cl_3_SiSiCl	HPL	3.56 × 10^19^	–1.48	24.3	2.52
		10 atm	6.35 × 10^19^	–2.56	15.5	0.628
		1 atm	8.80 × 10^16^	–1.97	13.0	0.534
		0.1 atm	9.37 × 10^14^	–1.62	11.3	0.458
R3-2	Cl_2_SiSiCl_2_ → SiCl_2_ + SiCl_2_	HPL	4.01 × 10^28^	–4.15	30.1	2.54
		10 atm	3.81 × 10^19^	–2.55	16.9	0.660
		1 atm	5.57 × 10^15^	–1.73	14.0	0.558
		0.1 atm	7.25 × 10^12^	–1.18	12.1	0.481
R3-5	SiCl_3_+SiCl → SICl_2_ + SiCl_2_	10 atm	1.63 × 10^10^	0.970	–0.515	0.340
		1 atm	1.64 × 10^10^	0.970	–0.515	0.129
		0.1 atm	1.64 × 10^10^	0.970	–0.515	0.129

a Activation energies are given in kilocalories per mole. HPL stands for high pressure limit.

The relative error of the Arrhenius parameter fitting for the recombination reactions tends to increase as the pressure drops. Reaction –R2-2 could not be fitted to the modified Arrhenius equation at 0.1 atm. All recombination reactions (–R1-1, –R1-8, –R1-2, –R1-7, –R1-12, and –R2-2) and some chemical activated reactions (R1-12, R1-13, R1-14, and R1-20), which have their maximum relative error exceeding 3% in Table 4, were also parameterized using the Chebyshev coefficients *a*_*ij*_ proposed by Venkatesh et al.^36^ and defined as
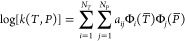
5where  are functions of *T* and *P*, respectively, and the Φ’s are Chebyshev polynomials of order *N*_*T*_ and *N*_*P*_. The coefficients in Table 5 were obtained using SSUMES by fitting eq 5 to the rate coefficients of all channels that should be considered in accurate modeling. These main channels were those whose branching ratios were at least 10% along the whole range of temperature from 873 to 1373 K and of pressure from 0.001 to 10 atm, respectively. The rate coefficients in this range can be obtained by the Chebyshev coefficients by using SSUMES^22^ or CHEMKIN.^24^*N*_*T*_ and *N*_*P*_ were set first to 5 and 4, respectively, to achieve small enough deviations (mean absolute deviations, MADs). Table 5 shows that the largest MAD, 0.02378, which resulted for –R2-2, corresponds to a mean deviation of 10^0.02378^ = 1.05628, or 5.63%.

**Table 5:** Chebyshev Coefficients (Six Significant Digits) Obtained From Fitting the Rate Coefficients Determined in This Work to eq r5^a^

		*N*_P_				
reaction	*N*_T_	1	2	3	4	log (MAD)
–R1-1SiH_2_Cl_2_ + SiCl_2_→ Cl_3_SiSiH_2_Cl	1	7.58833	0.8261	–0.15886	–0.00585	0.005873
	2	0.158888	0.566717	–0.03475	–0.02244	
	3	–0.18326	0.164868	0.007671	–0.00962	
	4	–0.07332	0.052381	0.012395	–0.00515	
	5	–0.02593	0.010501	0.010854	–0.00139	
–R1-8SiH_2_Cl_2_ + SiCl_2_ → HCl_2_SiSiHCl_2_	1	6.83497	0.834831	–0.16029	–0.00347	0.003572
	2	0.463168	0.481962	–0.03106	–0.01512	
	3	–0.1277	0.116851	–0.00245	–0.0046	
	4	–0.0464	0.03956	0.000934	–0.00343	
	5	–0.01628	0.011681	0.003567	–0.002	
R1-12SiH_2_Cl_2_ + SiCl_2_ → SiHCl_3_ + SiHCl	1	7.07892	–0.48718	–0.16394	–0.02613	0.002126
	2	1.15943	0.312074	0.061131	–0.00725	
	3	0.00602	0.004404	0.009218	0.001296	
	4	0.003855	0.003908	0.001313	–0.00053	
	5	0.001897	0.00322	0.001435	–0.00017	
R1-13SiH_2_Cl_2_ + SiCl_2_ → Cl_3_SiSiCl + H_2_	1	6.41258	–0.32931	–0.14488	–0.0336	0.002731
	2	1.31356	0.233478	0.075646	0.001732	
	3	0.007727	–0.00351	0.005859	0.003263	
	4	0.002727	0.002644	0.000925	–2.36 × 10^–5^	
	5	0.001408	0.002311	0.00119	0.00012	
R1-14SiH_2_Cl_2_ + SiCl_2_ → SiCl_4_ + SiH_2_	1	5.5124	–0.31217	–0.14131	–0.03416	0.002773
	2	1.33972	0.223433	0.076323	0.003222	
	3	0.007983	–0.00408	0.005145	0.003357	
	4	0.002622	0.00234	0.000805	–1.32 × 10^–5^	
	5	0.001321	0.002131	0.001105	0.000129	
–R1-2SiHCl_3_ + SiHCl → Cl_3_SiSiH_2_Cl	1	8.73054	1.00144	–0.19272	–0.00532	0.005901
	2	–0.27149	0.593014	–0.02846	–0.0231	
	3	–0.1868	0.162628	0.008228	–0.0092	
	4	–0.07331	0.052091	0.012239	–0.00518	
	5	–0.02591	0.01051	0.010849	–0.0014	
–R1-7SiHCl_3_ + SiHCl → HCl_2_SiSiHCl_2_	1	9.07598	0.716278	–0.13967	–0.00328	0.006272
	2	–0.14106	0.460778	–0.03528	–0.01473	
	3	–0.17716	0.118351	–0.00285	–0.0049	
	4	–0.02992	0.039786	0.001055	–0.00341	
	5	–0.00359	0.011672	0.003571	–0.00199	
–R1-12SiHCl_3_ + SiHCl → SiH_2_Cl_2_ + SiCl_2_	1	9.49174	–0.49287	–0.16296	–0.02551	0.004339
	2	0.643246	0.313673	0.059736	–0.00718	
	3	–0.02068	0.00449	0.008651	0.00115	
	4	0.010604	0.003017	0.001225	–0.00051	
	5	0.008857	0.002893	0.001287	–0.00017	
R1-20SiHCl_3_ + SiHCl → Cl_3_SiSiCl + H_2_	1	7.85438	–0.29788	–0.13577	–0.03395	0.002705
	2	0.865941	0.21334	0.07495	0.004889	
	3	0.007877	–0.00635	0.004111	0.003418	
	4	0.002048	0.001546	0.000445	3.73 × 10^–6^	
	5	0.001057	0.001698	0.000874	0.000115	
–R2-2SiCl_3_ + SiCl_3_ → Si_2_Cl_6_	1	5.72294	5.67814	–1.04715	0.020066	0.02378
	2	–1.23433	0.663641	0.26305	–0.06128	
	3	–0.41417	0.104118	0.082753	0.005193	
	4	–0.05121	–0.10996	0.038448	0.015586	
	5	0.017532	–0.06276	–0.0137	0.010007	

a Only channels that are expected to proceed with significant rates and that could not be fitted with the modified Arrhenius equation are listed (see the text). MAD stands for mean absolute deviation.

## 4 Conclusions

In this work, the elementary reactions connected to the potential energy wells of Si_2_H_2_Cl_4_, Si_2_Cl_6_, and Si_2_Cl_4_ were analyzed in detail. These species are important intermediates in CVD processes that use chlorinated monosilane as the silicon source gas. The pressure dependence of the rate coefficients for the unimolecular decomposition channels of these species and also of chemical activation reactions that proceed in the reverse direction relative to the decomposition channels were determined using the program suites GPOP^18^ and SSUMES.^22^ The structure optimizations and vibration frequencies were determined using Gaussian 09.^8^ Hindered rotor analysis for the torsion motion of the Si–Si bond was conducted, and accurate partition functions for this motion were obtained.

The results obtained in this work showed that the reactions investigated are in the fall-off regime under atmospheric or moderate pressure conditions and suggest that accurate modeling of CVD processes that employ chlorinated monosilanes as the silicon source requires careful determination of the rate coefficients as functions of temperature and pressure for the conditions of interest, instead of adopting high-pressure-limit rate coefficients.

Regarding the set of reactions r1–r8 pointed out as important in the Introduction section, SiHCl_3_ is converted to SiH_2_Cl_2_ via the intermediate Cl_3_SiSiH_2_Cl through reactions r4 (–R1-2: SiHCl_3_ + SiHCl → Cl_3_SiSiH_2_Cl) and r5 (R1-1: Cl_3_SiSiH_2_Cl → SiH_2_Cl_2_ + SiCl_2_) at high pressure. However, the recombination reaction r4 is less likely to occur at moderate or lower pressure; under these conditions, SiHCl_3_ is converted to SiH_2_Cl_2_ directly through reaction –R1-12 (SiHCl_3_ + SiHCl → SiH_2_Cl_2_ + SiCl_2_). At moderate pressure, the rate coefficient of r7 (R3-2: Cl_2_SiSiCl_2_ → SiCl_2_ + SiCl_2_) is about 2 or 3 orders of magnitude smaller than that at the high pressure limit; therefore, the decomposition of Si_2_Cl_4_ into SiCl_2_ becomes more difficult at moderate pressure. Reaction r8 (–R2-2: SiCl_3_ + SiCl_3_ → SiCl_6_) hardly occurs under moderate pressure; SiCl_3_ is converted to SiCl_2_ and SiCl_4_ through R2-3 (SiCl_3_ + SiCl_3_ → SiCl_4_ + SiCl_2_).

The rate coefficients calculated in this work for the important channels were parameterized through Chebyshev coefficients and fitted to the modified Arrhenius equation to be used in simulations of Si–H–Cl systems under a practical range of temperature and pressure.

Work on surface reactions is also in progress,^37−40^ and in near future, we intend to build a kinetic model that includes both gas-phase and surface reactions to allow accurate simulation of the CVD reaction.

## Supporting Information Available

Sensitivity of the total rate coefficient for the dissociation reactions to ⟨Δ*E*_down_⟩; the structures of the stable species, radicals, and tight TSs calculated at the CCSD(T)/CBS//B3LYP/6-31+G(d,p) level of theory; and hindered rotor analyses for the SiHCl_2_ rotor in HCl_2_SiSiHCl_2_, SiCl_3_ rotor in Si_2_Cl_6_, SiCl rotor in Cl_3_SiSiCl, SiHCl_2_ rotor in TS4, SiHCl_3_ rotor in TS6, and SiHCl_2_ rotor in TS11 (PDF)
