# Supplementary material for: Pressure Dependence of Rate Coefficients of Unimolecular and Chemical Activation Reactions Connected to the Potential Energy Wells of Si2H2Cl4, Si2Cl6, and Si2Cl4 via Rice–Ramsperger–Kassel–Marcus Calculations
Source: J Phys Chem A. 2022 Nov 13;126(46):8658–73. doi: 10.1021/acs.jpca.2c06195 (PMC9706569; doi:10.1021/acs.jpca.2c06195)
Supplement: Supplementary file 1 — jp2c06195_si_001.pdf [file jp2c06195_si_001.pdf]

Supporting Information for

Pressure dependence of rate coefficients of unimolecular and chemical activation reactions connected to the potential energy wells of $Si_2H_2Cl_4$, Si_2Cl_6, and Si_2Cl_4 via RRKM calculations

*Kaito Noda, Yoshihiro Jagawa, Akio Fuwa, and Nílson Kunioshi **

S1. Sensitivity of the total rate coefficient for the dissociation reactions to $\boldsymbol{\alpha_0}$.

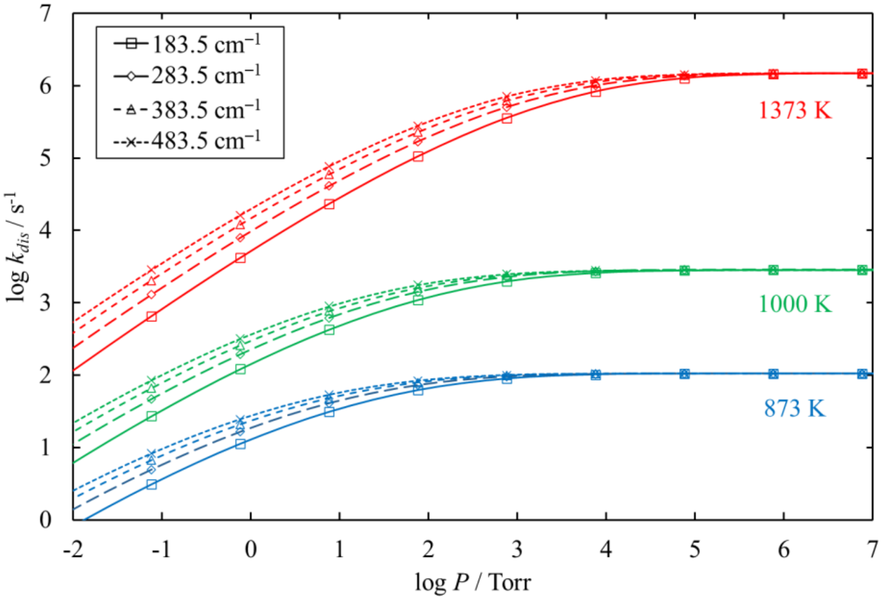

Figure S1 Sensitivity of the total rate coefficient for the dissociation of Cl_3SiSiH_2Cl to α_0 at 873, 1000, and 1373 K.

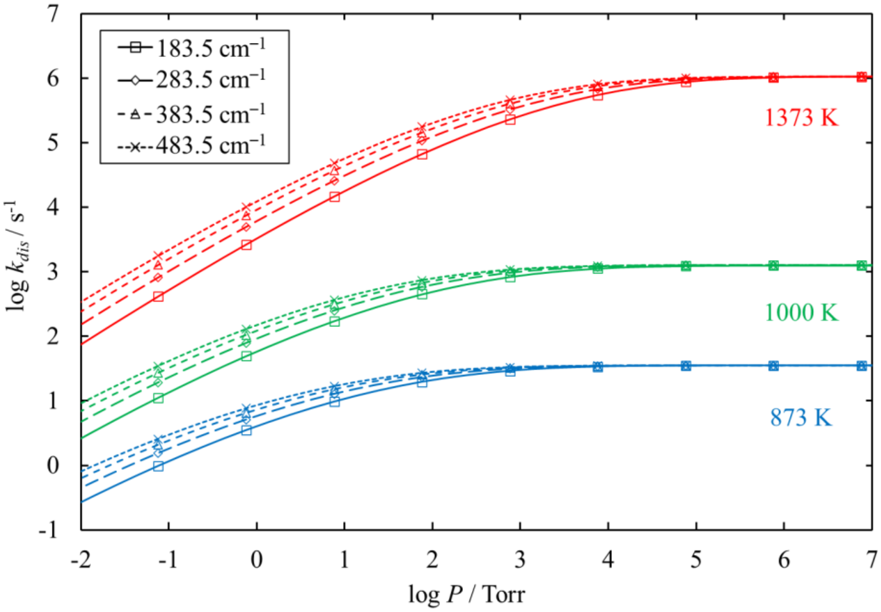

Figure S2 Sensitivity of the total rate coefficient for the dissociation of $HCl_2SiSiHCl_2$ to α_0 at 873, 1000, and 1373 K.

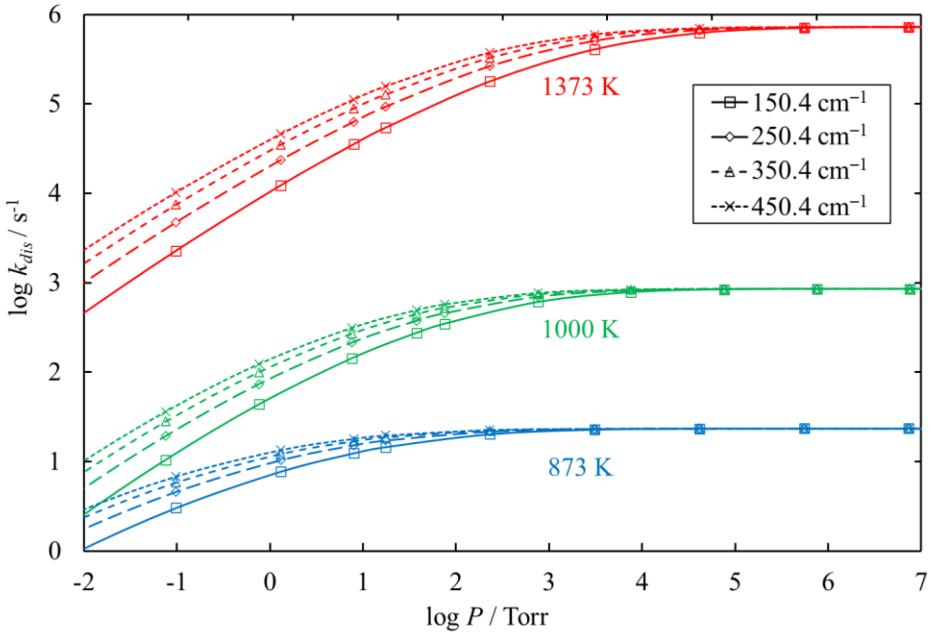

Figure S3. Sensitivity of the total rate coefficient for the dissociation of Si_2Cl_6 to α_0 at 873, 1000, and 1373 K.

S2. The structures of the species, radicals, and tight TSs calculated at the CCSD(T)/CBS//B3LYP/6-31+G(d,p) level of theory.

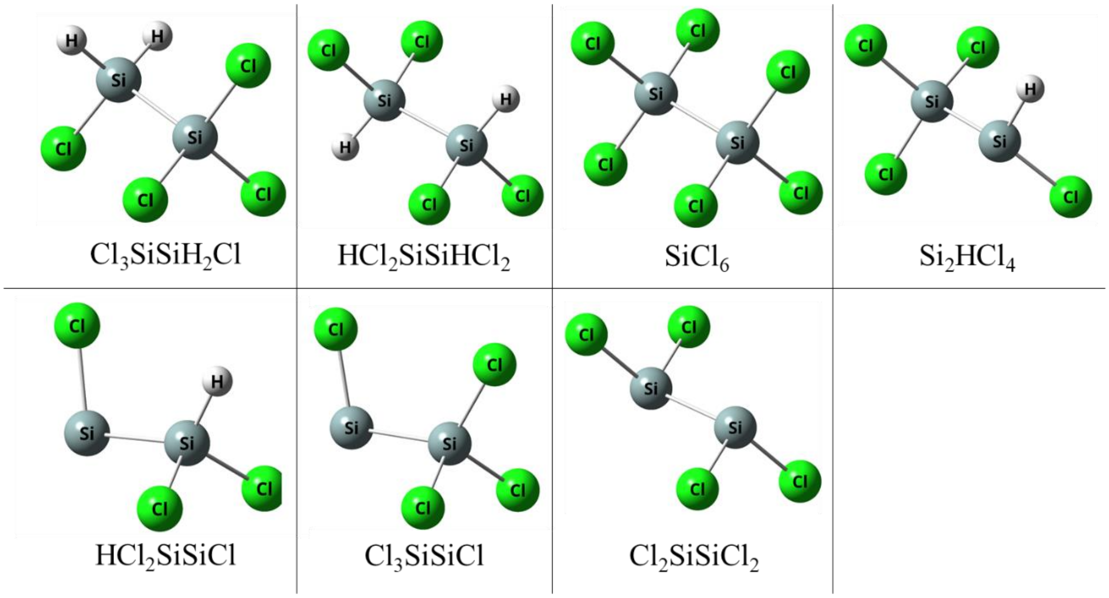

Figure S4. Structures of the stable species and radicals.

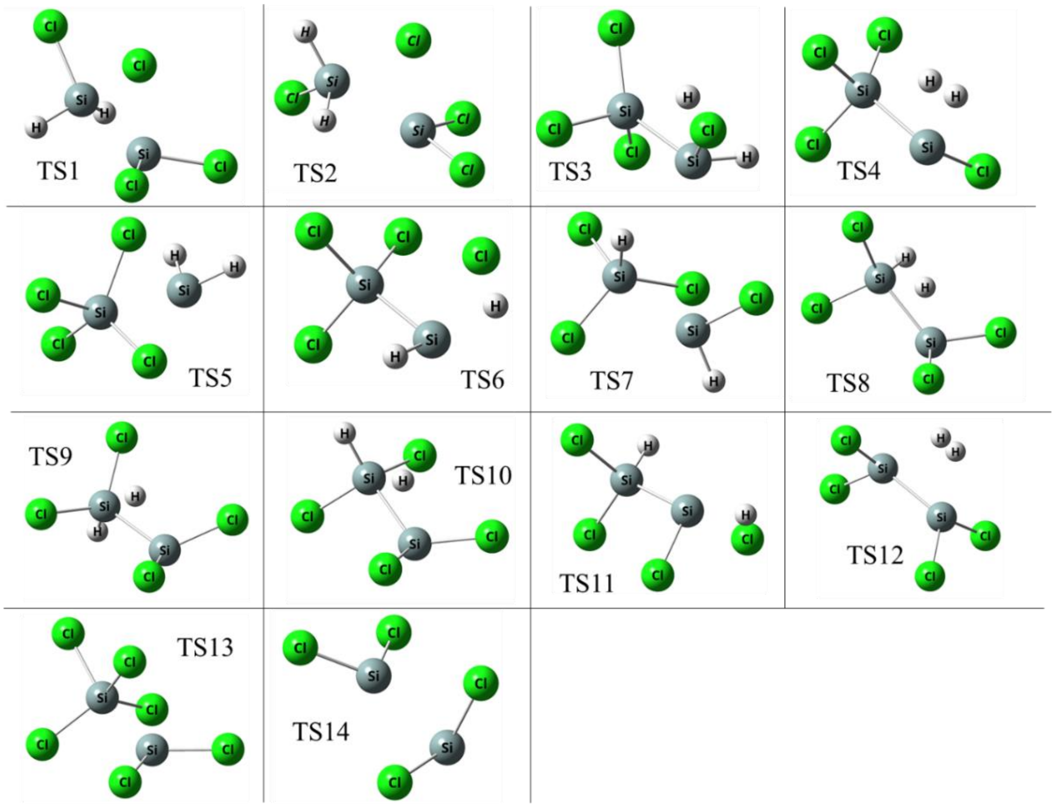

Figure S5. Structures of the optimized transition states.

Table S1. Cartesian coordinates calculated at B3LYP/6-31+G (d,p) level.

Species	Atomic type	Coordinates (Å)		
		X	Y	Z
Cl_3SiSiH_2Cl	Si	–1.426965	1.141155	0.000372
	Si	0.631785	0.000068	0.000004
	Cl	–2.998146	–0.217209	0.000147
	Cl	0.843754	–1.185094	1.678645
	Cl	2.145175	1.416879	–0.001280
	Cl	0.841925	–1.186478	–1.677876
	H	–1.511844	1.972462	1.221955
	H	–1.511669	1.972755	–1.221029
$HCl_2SiSiHCl_2$	Si	–1.071985	–0.000052	0.491148
	Si	1.071973	–0.000052	–0.491128
	Cl	–2.088519	–1.695924	–0.132872
	Cl	2.088541	–1.695908	0.132864
	Cl	2.088588	1.695981	0.132275
	H	–1.038523	–0.000301	1.967646
	H	1.038463	–0.000304	–1.967626
	Cl	–2.088597	1.695972	–0.132285
Si_2Cl_6	Si	–1.178146	–0.000070	0.000104
	Si	1.178178	–0.000100	0.000003
	Cl	–1.869469	1.864854	0.532638
	Cl	–1.869940	–0.471199	–1.881106
	Cl	–1.870382	–1.393500	1.348658

	Cl	1.870267	0.469548	1.881436
	Cl	1.869323	1.394724	–1.347585
	Cl	1.870174	–1.864288	–0.534129
Si_2HCl_4	Si	–1.435318	1.145066	–0.195133
	Si	0.618263	0.008785	–0.004161
	Cl	–3.037193	–0.163768	0.019278
	Cl	0.862436	–0.972697	1.801967
	Cl	2.113789	1.430765	–0.183920
	Cl	0.825166	–1.373589	–1.523525
	H	–1.552603	2.194003	0.855527
$HCl_2SiSiCl$	Si	0.588299	–0.000030	–0.429516
	Si	–1.348045	–0.000564	1.059018
	Cl	1.706442	–1.707651	0.031550
	H	0.402436	0.000019	–1.899738
	Cl	1.705393	1.708216	0.031801
	Cl	–2.809834	–0.000077	–0.470016
$Cl_3SiSiCl$	Si	–1.503517	–1.339132	–0.143578
	Si	0.549883	–0.005677	–0.011184
	Cl	–2.904004	0.219777	–0.021019
	Cl	1.459135	–0.551751	1.786697
	Cl	0.438240	2.062220	–0.070085
	Cl	1.791975	–0.622756	–1.568142
$Cl_2SiSiCl_2$	Si	–0.381625	1.160489	0.000000
	Si	0.381625	–1.160489	0.000000

	Cl	0.381625	2.160818	1.669091
	Cl	0.381625	2.160818	–1.669091
	Cl	–0.381625	–2.160818	1.669091
	Cl	–0.381625	–2.160818	–1.669091
TS1	Si	–1.655546	–0.850367	–0.217749
	Si	0.698061	0.222120	–0.347841
	Cl	–2.664303	0.947732	–0.571583
	Cl	2.371124	–0.791555	–1.074656
	Cl	–0.097283	–1.296005	1.423951
	Cl	1.415614	1.845754	0.738141
	H	–1.339146	–1.645035	–1.421392
	H	–2.683665	–1.560262	0.570142
TS2	Si	–1.449768	–0.000012	–0.821371
	Si	1.014850	0.000001	–0.418635
	Cl	–3.405283	0.000006	–0.026214
	Cl	2.203217	1.672240	–0.073719
	Cl	2.203232	–1.672228	–0.073719
	Cl	–0.475131	–0.000005	1.376072
	H	–1.426888	1.287489	–1.540552
	H	–1.426880	–1.287539	–1.540504
TS3	Si	–1.561692	1.001242	0.042028
	Si	0.644593	0.014802	–0.027848
	Cl	–3.138631	–0.347700	–0.274191
	Cl	0.651374	–1.153100	1.676480

	Cl	1.772080	1.729273	0.140533
	Cl	1.597706	–1.201377	–1.422322
	H	–0.490712	0.322983	–1.171503
	H	–1.672904	1.991780	–1.075489
TS4	Si	–0.612255	0.010942	–0.029245
	Si	1.500134	1.224431	0.023055
	Cl	–0.827757	–0.712676	1.899433
	Cl	–0.955242	–1.620701	–1.260226
	Cl	–2.057615	1.451118	–0.374968
	Cl	2.996329	–0.269891	–0.094925
	H	1.325047	1.606566	–1.490141
	H	0.597498	0.684757	–1.301553
TS5	Si	2.513626	–0.000109	–0.281793
	Si	–0.370358	–0.000026	–0.076324
	Cl	0.885753	0.000607	1.661003
	Cl	–0.412586	–1.756599	–1.143371
	Cl	–0.412591	1.755792	–1.144610
	Cl	–2.215157	0.000304	0.888067
	H	3.311019	–1.147815	0.287967
	H	3.311109	1.147933	0.287165
TS6	Si	1.170786	1.533539	0.086317
	Si	–0.643800	0.003414	–0.000963
	Cl	3.118770	–0.218377	–0.030495
	Cl	–0.645331	–1.327812	–1.587135

	Cl	–2.334034	1.210992	–0.161778
	Cl	–0.806236	–1.097227	1.746154
	H	1.249024	1.890178	–1.370311
	H	2.709304	0.943677	0.740679
TS7	Si	–0.843906	0.049635	–0.451593
	Si	1.569772	0.690255	0.225510
	Cl	–1.619188	1.862585	0.207986
	Cl	0.234529	–1.193048	1.059488
	Cl	3.273100	–0.197906	–0.598088
	H	–0.412759	–0.096944	–1.849040
	H	2.041791	1.128435	1.576579
	Cl	–2.582039	–1.141628	–0.467174
TS8	Si	1.097980	–0.512904	–0.244053
	Si	–1.162786	0.156774	–0.364657
	Cl	2.317250	–0.724793	1.435975
	Cl	–2.569957	–1.377510	–0.477238
	Cl	–1.887805	1.637284	0.904091
	H	0.965571	–1.794142	–0.951143
	H	–0.207516	–0.645008	0.834184
	Cl	2.149291	0.901782	–1.354658
TS9	Si	1.148228	0.014530	0.529120
	Si	–1.243217	–0.040280	0.491182
	Cl	2.130443	–1.722484	–0.072662
	Cl	–2.133978	–1.693708	–0.388041

	Cl	–1.930546	1.808521	–0.114201
	H	–1.662992	–0.167988	1.899874
	H	–0.069153	–0.083136	–0.715797
	Cl	2.114197	1.643649	–0.334997
TS10	Si	–1.227834	–0.101106	0.583710
	Si	0.979773	–0.096374	–0.322980
	Cl	–2.158155	–1.612869	–0.509045
	Cl	2.091120	1.665070	–0.262842
	Cl	2.213459	–1.668804	0.284543
	H	–1.709886	–0.401843	1.944456
	H	0.302012	0.144511	1.272996
	Cl	–1.859323	1.794370	0.083363
TS11	Si	–0.987520	–0.578856	–0.425432
	Si	0.941930	0.814913	–0.720034
	Cl	–2.627569	0.619185	–0.888188
	Cl	2.628729	–1.257056	–0.556958
	Cl	1.141962	1.875594	1.056749
	H	–0.992352	–1.712883	–1.373518
	H	2.338729	0.044902	–1.305386
	Cl	–1.184776	–1.334006	1.489304
TS12	Si	–1.197346	0.302645	0.418543
	Si	1.196940	–0.303063	0.418183
	Cl	–2.272727	–1.485618	0.587120
	Cl	2.271184	1.485753	0.588018

	Cl	2.178217	–1.410577	–1.061398
	H	–0.346275	0.255312	2.202774
	H	0.347153	–0.257248	2.202551
	Cl	–2.176391	1.410900	–1.061944
TS13	Si	1.059288	0.097274	–0.203128
	Si	–1.591333	–0.541023	–0.811102
	Cl	2.026383	1.688058	0.721518
	Cl	2.528438	–1.325314	–0.567694
	Cl	–2.359627	1.425211	–0.472697
	Cl	–1.757040	–1.422515	1.154122
TS14	Si	–1.413077	–0.000204	–0.263418
	Si	1.130941	–0.000034	–0.100592
	Cl	2.973120	–0.000511	0.894242
	Cl	1.278520	–1.774006	–1.169183
	Cl	1.278035	1.774932	–1.167508
	Cl	–2.623725	–1.665809	–0.039200
	Cl	–0.050484	–0.000232	1.821256
	Cl	–2.623118	1.665822	–0.039834

Table S2. Vibrational frequencies (v), rotational constants (B), symmetry numbers (σ) and point groups of the stable species and transition states The optimization and frequency calculation are at the B3LYP/6-31+G(d,p) level of theory.

Species	v (cm^{-1})	B (GHz)	σ (point group)
Cl_3SiSiH_2Cl	33.4, 63.8, 106, 135, 178, 184, 222, 358,487, 515, 559, 580, 584, 696, 829, 936, 2261, 2279	1.22, 0.634, 0.622	1 (C_s)
$HCl_2SiSiHCl_2$	22.5, 61.4, 124, 127, 156, 185, 227, 426, 494, 552, 556, 571, 699, 783, 788, 821, 2281, 2282	1.19, 0.722, 0.468	2 (C_{2h})
Si_2Cl_6	21.9, 64.9, 65.0, 123, 123, 129, 174, 174, 211, 211, 242, 341, 450, 579, 580, 592, 592, 611	0.640, 0.419, 0.419	6 (D_{3d})
Cl_3SiSiH	35.8, 99.4, 169, 173, 196, 234, 335, 506, 543, 559, 676, 2066	1.37, 1.19, 1.13	1 (C_s)
$HCl_2SiSiCl$	5.89, 81.2, 100, 129, 202, 412, 490, 506, 534, 729, 775, 2248	2.01, 0.859, 0.679	1 (C_s)
$Cl_3SiSICl$	4.05, 73.9, 79.4, 127, 171, 172, 224, 334, 503, 523, 542, 572	1.19, 0.666, 0.644	1 (C_s)
$Cl_2SiSiCl_2$	38.2, 55.2, 98.9, 111, 133, 188, 221, 280, 474, 523, 535, 538	1.24, 0.668, 0.447	2 (C_{2h})
$SiHCl_3$	172, 172, 248, 479, 584, 584, 806, 806, 2330	2.39, 2.39, 1.28	3 (C_{3v})
SiH_2Cl_2	184, 510, 570, 592, 712, 885, 958, 2286, 2304	14.0, 2.46, 2.15	2 (C_{2v})
$SiCl_4$	144, 144, 217, 217, 217, 407, 603, 603, 603	1.29, 1.29, 1.29	12 (T_d)
SiH_2Cl	525, 649, 731, 924, 2185, 2230	129, 6.86, 6.67	1 (C_s)
$SiHCl_2$	174, 493, 553, 652, 749, 2180	15.7, 2.45, 2.15	1 (C_s)
$SiCl_3$	161, 161, 234, 450, 560, 560	2.39, 2.39, 1.26	3 (C_{3v})
SiH_2	1030, 2039, 2042	236, 209, 111	2 (C_{2v})

SiHCl	495, 814, 2022	224, 7.10, 6.88	1 (C_s)
$SiCl_2$	190, 488, 495	14.4, 2.68, 2.26	2 (C_{2v})
SiCl	502	7.34	1 ($C_{\infty v}$)
H_2	4465	1818, 1818	2 ($D_{\infty v}$)
HCl	2943	312, 312	1 ($C_{\infty v}$)
TS1	205i, 45.5, 74.7, 110, 119, 145, 211, 266, 347, 503, 526, 539, 606, 673, 928, 989, 2264, 2273	1.24, 0.662, 0.579	1 (C_1)
TS2	229i, 42.2, 92.3, 128, 135, 159, 202, 265, 355, 433, 472, 548, 560, 793, 866, 887, 2254, 2300	1.72, 0.538, 0.484	1 (C_s)
TS3	189i, 51.1, 61.8, 135, 160, 192, 208, 249, 410, 498, 529, 538, 602, 691, 787, 1257, 1496, 2171	1.22, 0.619, 0.575	1 (C_1)
TS4	1082i, 37.2, 60.4, 131, 140, 186, 190, 209, 271, 446, 493, 547, 562, 591, 720, 1027, 1601, 1928	1.20, 0.645, 0.613	1 (C_1)
TS5	183i, 78.2, 105, 117, 181, 211, 211, 251, 266, 360, 497, 517, 535, 609, 631, 988, 2109, 2132	1.16, 0.800, 0.800	1 (C_s)
TS6	552i, 29.2, 47.6, 104, 109, 176, 180, 200, 292, 354, 494, 528, 563, 568, 597, 766, 1503, 2162	1.19, 0.613, 0.592	1 (C_1)
TS7	146i, 47.7, 75.4, 108, 155, 187, 239, 255, 381, 462, 509, 528, 599, 660, 778, 900, 2175, 2294	1.65, 0.582, 0.492	1 (C_1)
TS8	279i, 40.0, 67.5, 105, 140, 202, 237, 304, 477, 513, 548, 570, 612, 751, 838, 1081, 1526, 2319	1.27, 0.528, 0.507	1 (C_1)
TS9	317i, 12.1, 62.3, 111, 141, 158, 192, 325, 464, 545, 564, 584, 723, 818, 832, 1068, 1489, 2259	1.14, 0.706, 0.459	1 (C_s)
TS10	360i, 24.0, 67.0, 117, 153, 169, 192, 274, 462, 514, 545, 567, 588, 707, 842, 1208, 1478, 2278	1.17, 0.709, 0.469	1 (C_1)
TS11	723i, 21.4, 52.8, 76.7, 83.2, 120, 175, 248, 342, 414, 508, 546, 566, 636, 750, 785, 1389, 2271	1.09, 0.620, 0.549	1 (C_1)
TS12	433i, 48.1, 50.1, 70.6, 98.0, 129.2, 187.2, 205.9, 408.4, 451.3, 470.6, 516.2, 519.7, 578.5, 579.2, 1062.1, 1116.3, 2481.1	1.20, 0.564, 0.472	1 (C_s)

TS13	154i, 38.0, 52.4, 81.9, 108, 128, 151, 167, 215, 217, 235, 265, 340, 461, 511, 555, 556, 585	0.770, 0.358	0.408,	1 (C_s)
TS14	48.9i, 14.0, 29.6, 49.3, 108, 168, 194, 215, 439, 462,516, 538	1.22, 0.466	0.577,	1 (C_1)

S3. Hindered rotor analyses

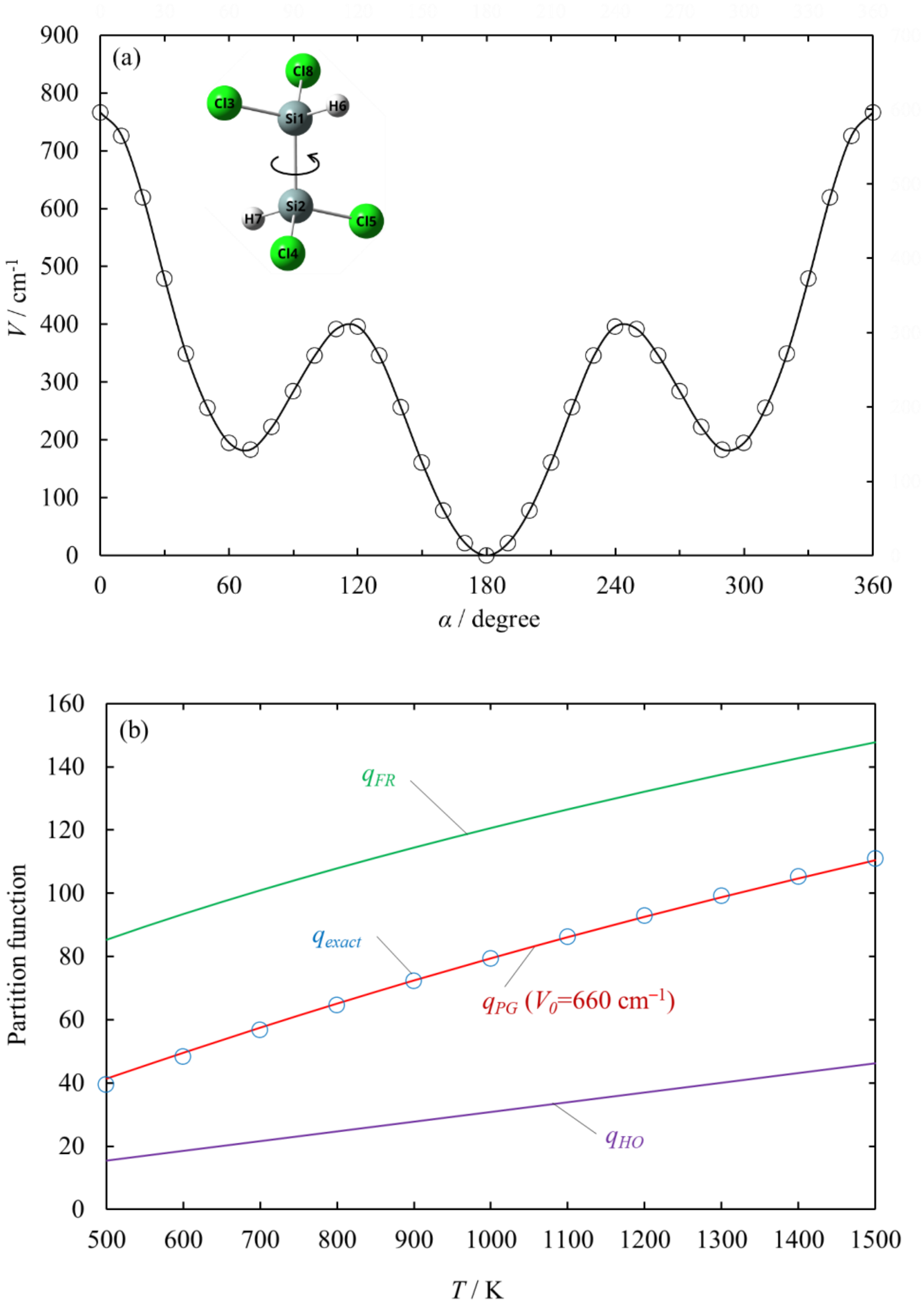

Figure S6. Hindered rotor analysis for $SiHCl_2$ rotor in $HCl_2SiSiHCl_2$: (a) Potential energy curve torsional angle α is defined as the dihedral angle between atoms H6-Si1-Si2-H7. The solid curve represents Fourier-series interpolation. (b) partition function calculated from eigenstate energies (q_{exact}) in comparison with harmonic oscillator (q_{HO}), free rotor (q_{FR}), and Pitzer-Gwinn (q_{PG}) approximations.

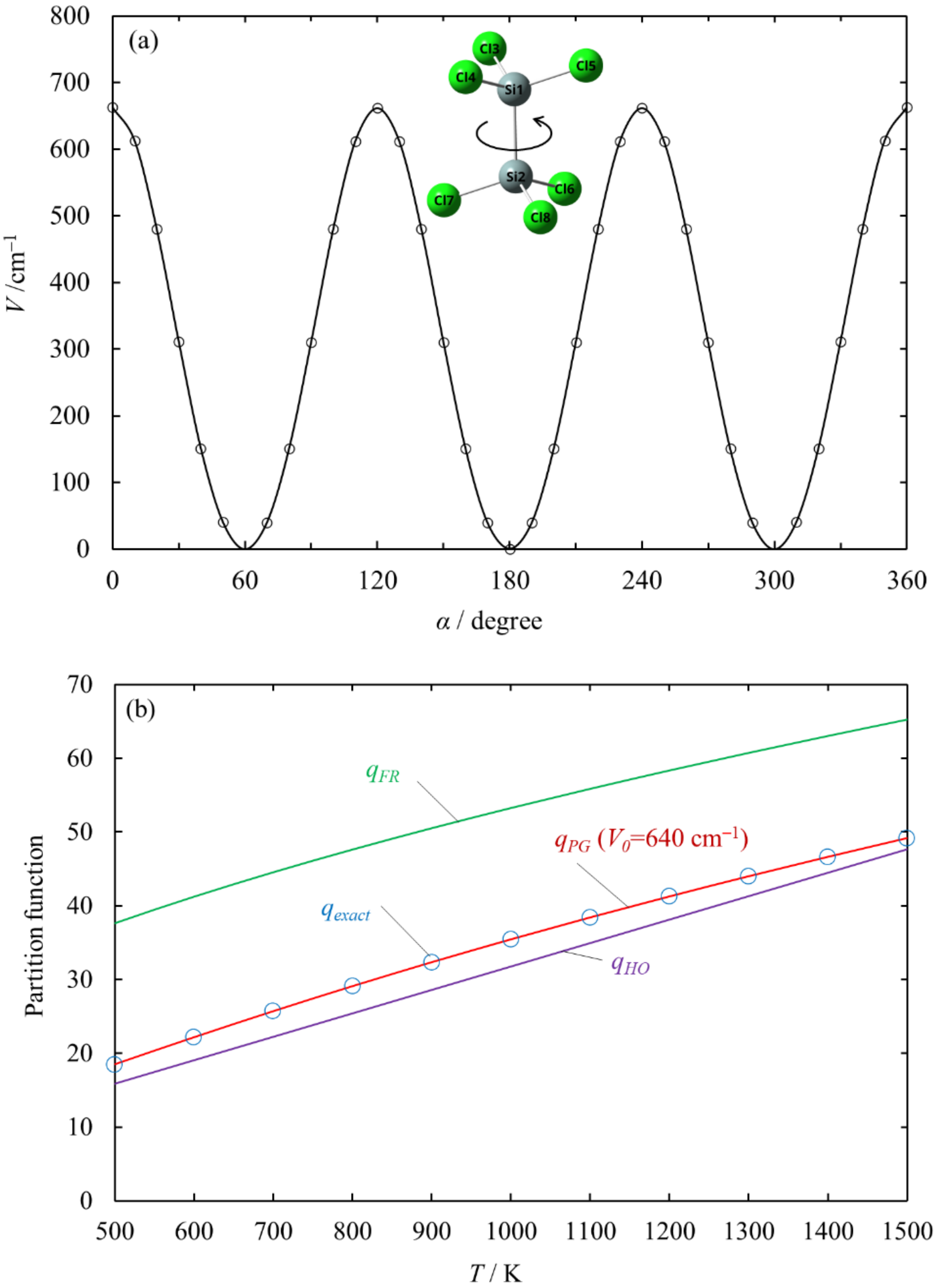

Figure S7. Hindered rotor analysis for $SiCl_3$ rotor in Si_2Cl_6: (a) Potential energy curve torsional angle α is defined as the dihedral angle between atoms Cl3-Si1-Si2-Cl6. The solid curve represents Fourier-series interpolation. (b) partition function calculated from eigenstate energies (q_{exact}) in comparison with harmonic oscillator (q_{HO}), free rotor (q_{FR}), and Pitzer-Gwinn (q_{PG}) approximations.

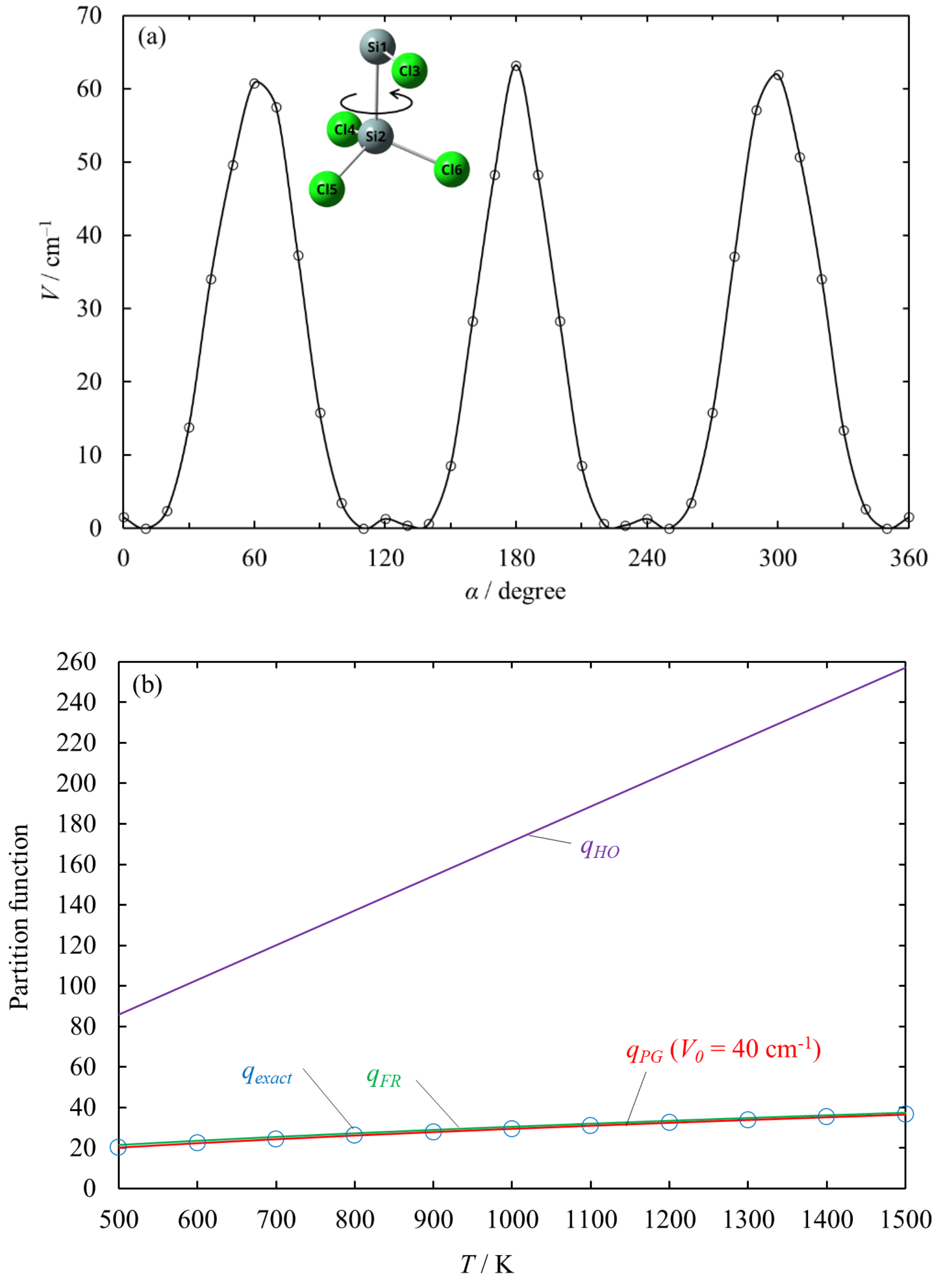

Figure S8. Hindered rotor analysis for SiCl rotor in $Cl_3SiSiCl$: (a) Potential energy curve torsional angle α is defined as the dihedral angle between atoms Cl3-Si1-Si2-Cl4. The solid curve represents Fourier-series interpolation. (b) partition function calculated from eigenstate energies (q_{exact}) in comparison with harmonic oscillator (q_{HO}), free rotor (q_{FR}), and Pitzer-Gwinn (q_{PG}) approximations.

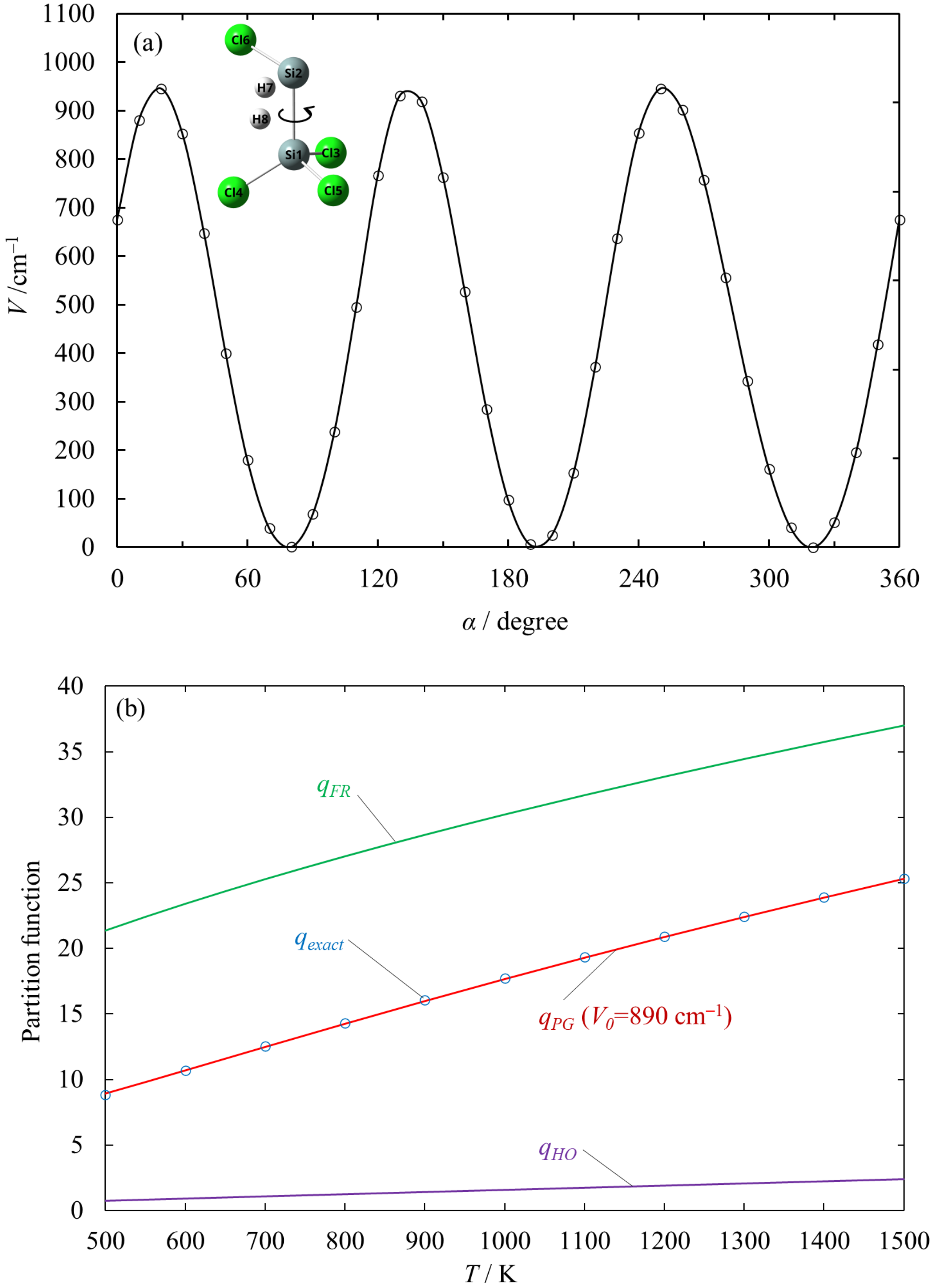

Figure S9. Hindered rotor analysis for $SiHCl_2$ rotor in TS4, which is TS of R1-3: (a) Potential energy curve torsional angle α is defined as the dihedral angle between atoms Cl3-Si1-Si2-Cl4. The solid curve represents Fourier-series interpolation. (b) partition function calculated from eigenstate energies (q_{exact}) in comparison with harmonic oscillator (q_{HO}), free rotor (q_{FR}), and Pitzer-Gwinn (q_{PG}) approximations.

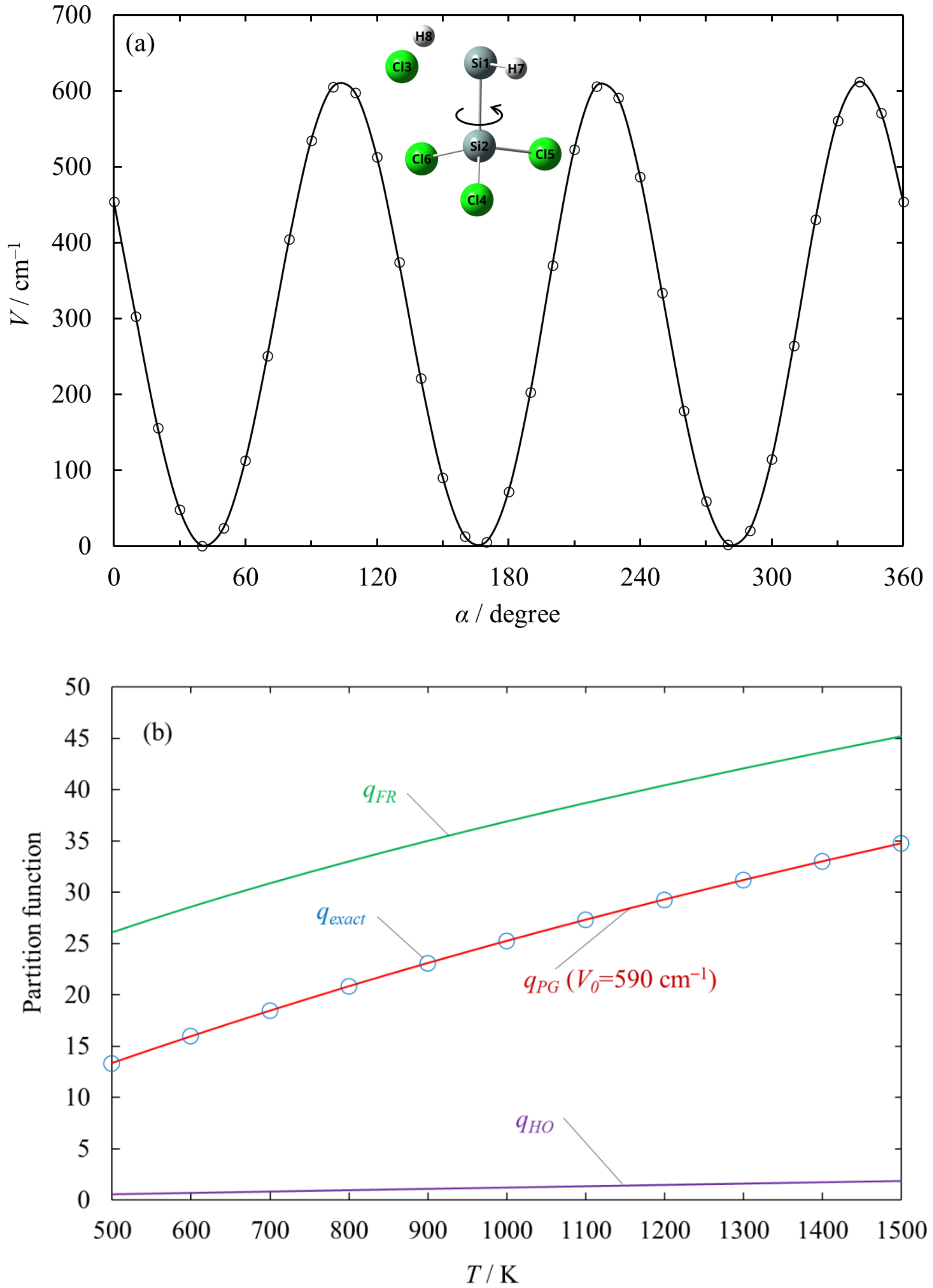

Figure S10. Hindered rotor analysis for $SiCl_3$ rotor in TS6, which is TS of R1-5: (a) Potential energy curve torsional angle α is defined as the dihedral angle between atoms H7-Si1-Si2-Cl4. The solid curve represents Fourier-series interpolation. (b) partition function calculated from eigenstate energies (q_{exact}) in comparison with harmonic oscillator (q_{HO}), free rotor (q_{FR}), and Pitzer-Gwinn (q_{PG}) approximations.

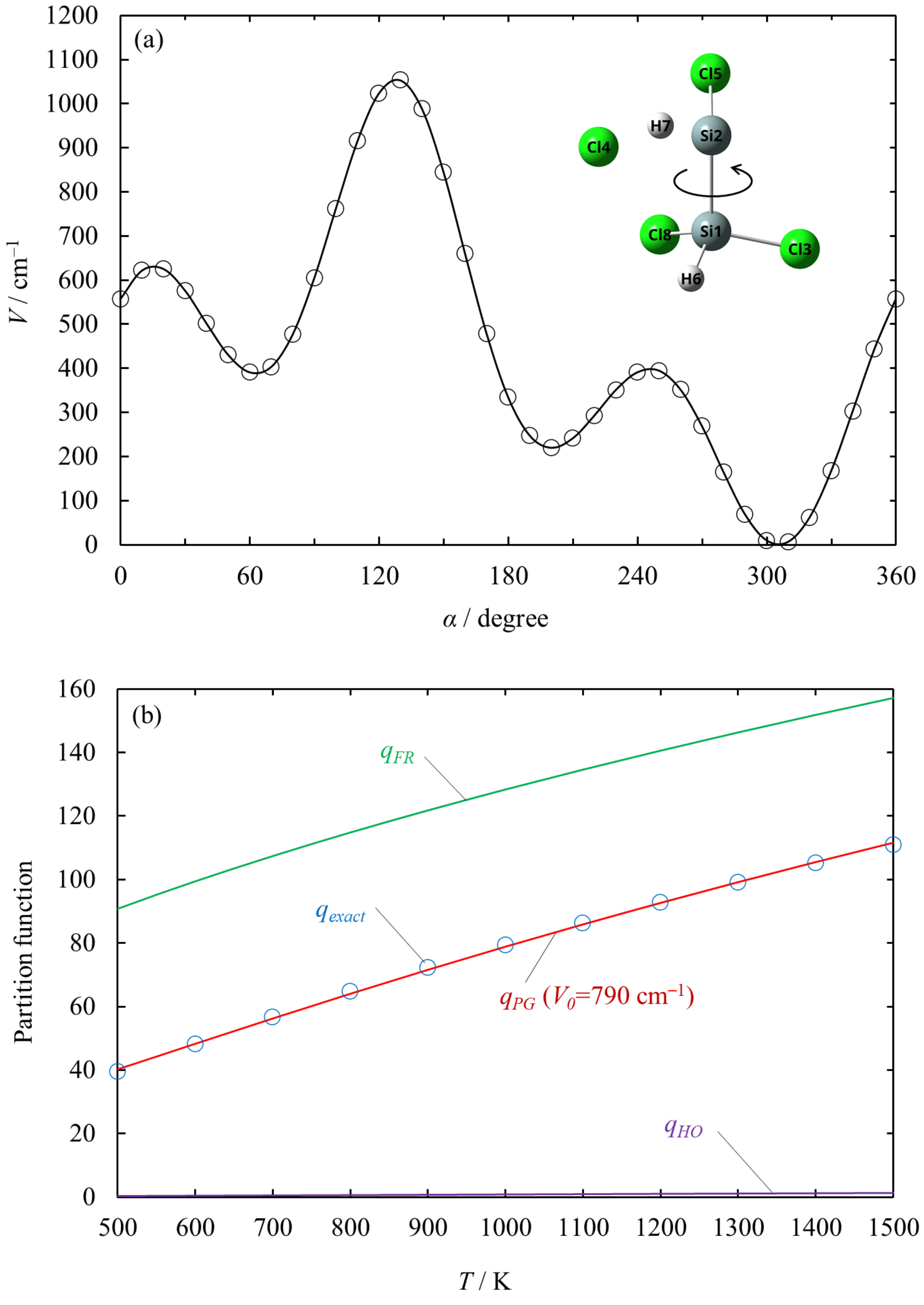

Figure S11. Hindered rotor analysis for $SiHCl_2$ rotor in TS11, which is TS of R1-10: (a) Potential energy curve torsional angle α is defined as the dihedral angle between atoms H7-Si1-Si2-Cl4. The solid curve represents Fourier-series interpolation. (b) partition function calculated from eigenstate energies (q_{exact}) in comparison with harmonic oscillator (q_{HO}), free rotor (q_{FR}), and Pitzer-Gwinn (q_{PG}) approximations.
